# The Effect of Tetanus-Diphtheria-Acellular-Pertussis Immunization During Pregnancy on Infant Antibody Responses: Individual-Participant Data Meta-Analysis

**DOI:** 10.3389/fimmu.2021.689394

**Published:** 2021-07-06

**Authors:** Bahaa Abu-Raya, Kirsten Maertens, Flor M. Munoz, Petra Zimmermann, Nigel Curtis, Scott A. Halperin, Nynke Rots, Daan Barug, Beth Holder, Beate Kampmann, Elke Leuridan, Manish Sadarangani

**Affiliations:** ^1^ Vaccine Evaluation Center, BC Children’s Hospital Research Institute, University of British Columbia, Vancouver, BC, Canada; ^2^ Department of Pediatrics, University of British Columbia, Vancouver, BC, Canada; ^3^ Centre for the Evaluation of Vaccination, Vaccine and Infectious Diseases Institute, University of Antwerp, Antwerp, Belgium; ^4^ Departments of Pediatrics and Molecular Virology and Microbiology, Baylor College of Medicine, Houston, TX, United States; ^5^ Department of Paediatrics, The University of Melbourne and Infectious Diseases Research Group, Murdoch Children’s Research Institute, Royal Children’s Hospital Melbourne, Parkville, VIC, Australia; ^6^ Department of Pediatrics, Fribourg Hospital HFR and Faculty of Science and Medicine, University of Fribourg, Fribourg, Switzerland; ^7^ Canadian Center for Vaccinology, Departments of Pediatrics and Microbiology and Immunology, Dalhousie University, Izaak Walton Killam Health Centre, and Nova Scotia Health Authority, Halifax, NS, Canada; ^8^ Centre for Infectious Disease Control, National Institute for Public Health and the Environment, Bilthoven, Netherlands; ^9^ Department of Metabolism, Digestion and Reproduction, Institute of Reproductive and Developmental Biology, Imperial College, London, United Kingdom; ^10^ Section of Paediatrics, Division of Infectious Diseases, Department of Medicine, Imperial College, London, United Kingdom; ^11^ Vaccines and Immunity Theme, Medical Research Council Unit The Gambia at the London School of Hygiene and Tropical Medicine, Fajara, Gambia; ^12^ The Vaccine Centre, Faculty of Infectious and Tropical Diseases, London School of Hygiene and Tropical Medicine, London, United Kingdom

**Keywords:** pertussis, immunization, pregnancy, infants, gestational

## Abstract

**Background:**

Immunization with tetanus-diphtheria-acellular pertussis (Tdap) vaccine in pregnancy is increasingly recommended. We determined the effect of Tdap immunization in pregnancy on infants’ vaccine responses.

**Methods:**

Individual-participant data meta-analysis of ten studies (n=1884) investigating infants’ antibody response to routine immunizations following Tdap immunization in pregnancy was performed. Geometric mean ratios (GMRs) of antigen-specific immunoglobulin G (IgG) levels were calculated using mixed-effects models. Seroprotection rates were compared using chi-squared tests.

**Results:**

Infants of Tdap-immunized women had significantly lower IgG against pertussis toxin (GMR 0.65; 95%CI 0.57-0.74), filamentous haemagglutinin (FHA) (0.68; 0.53-0.87), pertactin (0.65; 0.58-0.72) and fimbria 2/3 (FIM2/3) (0.41; 0.32-0.52) after primary immunization, compared with infants of unimmunized women. These lower levels persisted after booster immunization for FHA (0.72; 0.61-0.84) and FIM2/3 (0.53; 0.29-0.96). After primary immunization, infants of Tdap-immunized women had lower seroprotection rates against diphtheria (90% [843/973] *vs* 98% [566/579]; p<0.001) and invasive pneumococcal disease (IPD) caused by 5 *Streptococcus pneumoniae* (SPN) serotypes (SPN5, SPN6B, SPN9V, SPN19A, SPN23F), and higher seroprotection rates against *Haemophilus influenzae* type b (short-term and long-term seroprotection rates, 86%[471/547] *vs* 76%[188/247] and 62%[337/547] *vs* 49%(121/247), respectively, all p=0.001). After booster immunization, seroprotection rates against diphtheria and tetanus were 99% (286/288) and (618/619) in infants of Tdap-immunized women, respectively.

**Conclusions:**

Infants of Tdap-immunized women in pregnancy had lower IgG levels against pertussis, diphtheria and some SPN serotypes after their immunization compared with infants of unimmunized women. Enhanced surveillance of pertussis, diphtheria and IPD in infants is needed to determine the clinical significance of these findings.

**Systematic Review Registration:**

CRD42017079171.

## Introduction

Pertussis disease is caused in humans mainly by *Bordetella pertussis*, a gram-negative, aerobic coccobacillus bacterium (1). Clinical manifestations are divided into three classical stages: catarrhal, paroxysmal and convalescent. The catarrhal stage is an influenza-like disease with low-grade fever, malaise, nasal congestion, rhinorrhea, sneezing and mild cough. The paroxysmal stage is characterized by the classical “whooping cough” [many violent and rapid coughs followed by a high-pitch “whoop” voice (1)], which might be associated with vomiting. The convalescent stage is characterized by a decrease in paroxysmal cough frequency. Each stage lasts ~1-3 weeks (1). Pertussis is most severe in youngest infants leading to substantial morbidity and mortality (2, 3). Infants with pertussis can have severe complications such as apnea, seizures [reported in 3% of infants <30 days (4)].

Current infants’ and adults’ immunization programs in most high-income countries use acellular pertussis (aP) vaccines in their schedules. The aP vaccines are composed of purified bacterial antigens (pertussis toxin [PT], filamentous hemagglutinin [FHA], pertactin [PRN], and some aP vaccines has also fimbriae [FIM2/3]).

Immunization with tetanus-diphtheria-acellular pertussis (Tdap) vaccine in pregnancy has been implemented in an increasing number of countries leading to successful reduction in pertussis incidence, morbidity and mortality in young infants (5–7). While the mechanism of protection *via* immunization in pregnancy has not been established, it is mediated, at least in part, by increasing anti-*B. pertussis* antibodies in the newborn (8, 9). Early studies in infants born to women not immunized in pregnancy suggested that higher pre-existing maternally derived antibody levels could have a suppressive effect on infants’ active immune responses to their own immunizations leading to low post-immunization antibody levels (10–13). Later studies in infants born to women immunized with Tdap in pregnancy showed modification of immune responses to immunizations in infancy, leading to lower antibody levels in infants born to women immunized with Tdap compared with infants born to women unimmunized in pregnancy (8, 14–16). However, data are conflicting regarding the antigen-specific antibodies affected, the degree, quantity and the duration of such modifications in immune responses.

While the focus of these analyses has been on pertussis-specific antibody responses, Tdap vaccines administered in pregnancy also include tetanus and diphtheria antigens – which may thus influence responses to the same antigens in infants, as well as protein-polysaccharide conjugate vaccines (such as *Haemophilus influenzae* type b [Hib] and pneumococcal) which include these antigens as carrier proteins. Data are lacking on whether these immune effects translate into lower seroprotection rates for diseases in which correlates of protection exist (tetanus, diphtheria, Hib and invasive pneumococcal disease [IPD]). The aim of this study was to determine how Tdap immunization in pregnancy modifies infants’ antibody response to their own routine primary and booster immunizations and affects seroprotection rates.

## Methods

### Search Strategy and Selection Criteria

This study followed Preferred Reporting Items for Systematic Reviews and Meta-Analyses (PRISMA) for Individual-Patient Data reporting guidelines (**Supplementary Table 1**) (17). PubMed, MEDLINE, Embase, Cumulative Index to Nursing and Allied Health Literature (CINAHL), and the Cochrane Central Register of Controlled Trials (CENTRAL) databases were searched for English literature reporting immunoglobulin G (IgG) levels following primary and booster immunizations in infants born to women immunized against pertussis in pregnancy and infants of women unimmunized in pregnancy, published between January 1^st^, 1990 and January 6^th^, 2020 (**Supplementary Methods**).

Studies were included if they:

1) Measured IgG levels to at least one of the following antigens – PT, FHA, PRN, FIM2/3, tetanus-toxoid [TT], diphtheria-toxoid [DT]), Hib polyribosyl ribitol phosphate (PRP), *Neisseria meningitidis* or *Streptococcus pneumoniae* (SPN) – in infants post-primary and/or post-booster (at age 9-24 months) immunizations with diphtheria-tetanus-acellular pertussis (DTaP), Hib, meningococcal conjugate and/or pneumococcal conjugate vaccines (PCV).
2) Reported these IgG levels for infants born at ≥36 weeks gestation (WG) to women immunized at any time in pregnancy with a single dose of Tdap vaccine and for infants born at ≥36 WG to women unimmunized with Tdap in pregnancy in the same study (the use of TT or diphtheria and tetanus toxoids (dT) vaccines in pregnancy as a control to Tdap immunization in pregnancy was allowed).

Studies were excluded if they:

1) Included only infants <36 WG;
2) Included only infants or women with an immunologic disorder;
3) Included only infants who received immunoglobulins in the previous year before antibody response assessment;
4) Included only women who received immunosuppressive drugs during the current pregnancy, blood products 3 months prior to delivery, intravenous immunoglobulins within the previous year before delivery, immunosuppressive drugs or blood products 3 months prior to antibody response assessment in the women.

Authors of the identified studies were contacted to share individual-participant data for the meta-analysis.

### Data Analysis

An individual-participant data meta-analysis of anti-*B. pertussis* antibody levels in infants of women immunized with Tdap in pregnancy compared with infants of women unimmunized with Tdap in pregnancy was done. Anti-FIM2/3 antibody analyses were restricted to women and infants who received FIM2/3-containing vaccines in pregnancy and in infancy, respectively. For anti-TT and anti-PRP antibody analyses, women unimmunized with Tdap but immunized with TT or dT in pregnancy were excluded from analyses, as well their infants. For anti-DT and anti-SPN antibody analyses, women unimmunized with Tdap but immunized with dT in pregnancy were excluded from analyses, as well their infants.

In order to ensure individual-participant data integrity, data received were recapitulated for each study for main baseline characteristics and IgG levels and compared with numbers of participants and data reported by the authors.

IgG levels were log2-transformed and meta-analyzed using mixed-effects models for each antigen-specific antibody and time point with study site and primary vaccination schedule as random intercepts. The main time points were post-primary and post-booster immunization in infants. Additional analysis time points were: pre-immunization in pregnancy, post-immunization in pregnancy (4 weeks after immunization in pregnancy), at delivery (maternal and cord blood), pre-primary immunization and pre-booster immunization. The mixed-effects models included co-variates known to influence immune responses to immunization and were available within the datasets (18). For the maternal time points and infants’ time points pre-primary immunization, adjustment was made for maternal age at immunization and pre-existing homologous antibody levels. For the infants’ post-primary, pre-booster and post-booster immunization time points, adjustment was made for infant sex and infant age at primary immunization.

The antilog (2*x*) of the coefficients from the models and their 95% confidence intervals (CIs) were presented as geometric mean ratios (GMRs) and their 95% CIs. For maternal time points, the GMR was interpreted as the ratio of antigen-specific IgG levels in women immunized with Tdap in pregnancy *versus* levels in women unimmunized in pregnancy. For infants’ time points, the GMR was interpreted as the ratio of antigen-specific IgG levels in infants born to women immunized with Tdap in pregnancy *versus* levels in infants of women unimmunized in pregnancy.

Seroprotection rates against tetanus disease (anti-TT IgG ≥0.1 IU/mL), diphtheria disease (anti-DT IgG ≥0.1 IU/mL), IPD (anti-SPN IgG ≥0.35 μg/mL), Hib disease (anti-PRP IgG ≥0.15 µg/ml and anti-PRP IgG ≥1 µg/ml for short- and long- term protection, respectively) were calculated (19). A chi-squared test was used to determine whether the seroprotection rates were different among women immunized with Tdap in pregnancy compared with unimmunized women, and in infants born to women immunized with Tdap in pregnancy compared with infants of unimmunized women.

Risk of bias of randomized-controlled trials was assessed against the Cochrane Risk of Bias tool for randomized-controlled trials. Risk of bias of non-randomized studies was assessed using ROBIN-I tool (20). Risk of bias was assessed by two independent researchers (Bahaa Abu-Raya and Kirsten Maertens).

R version 3.4.0 was used for all analysis (meta package, version 4.9-1). The study was registered at The International prospective register of systematic reviews PROSPERO (CRD42017079171).

## Role of the funding source

The funders had no role in the design, analysis or interpretation of the results.

## Results

**Figure 1:**
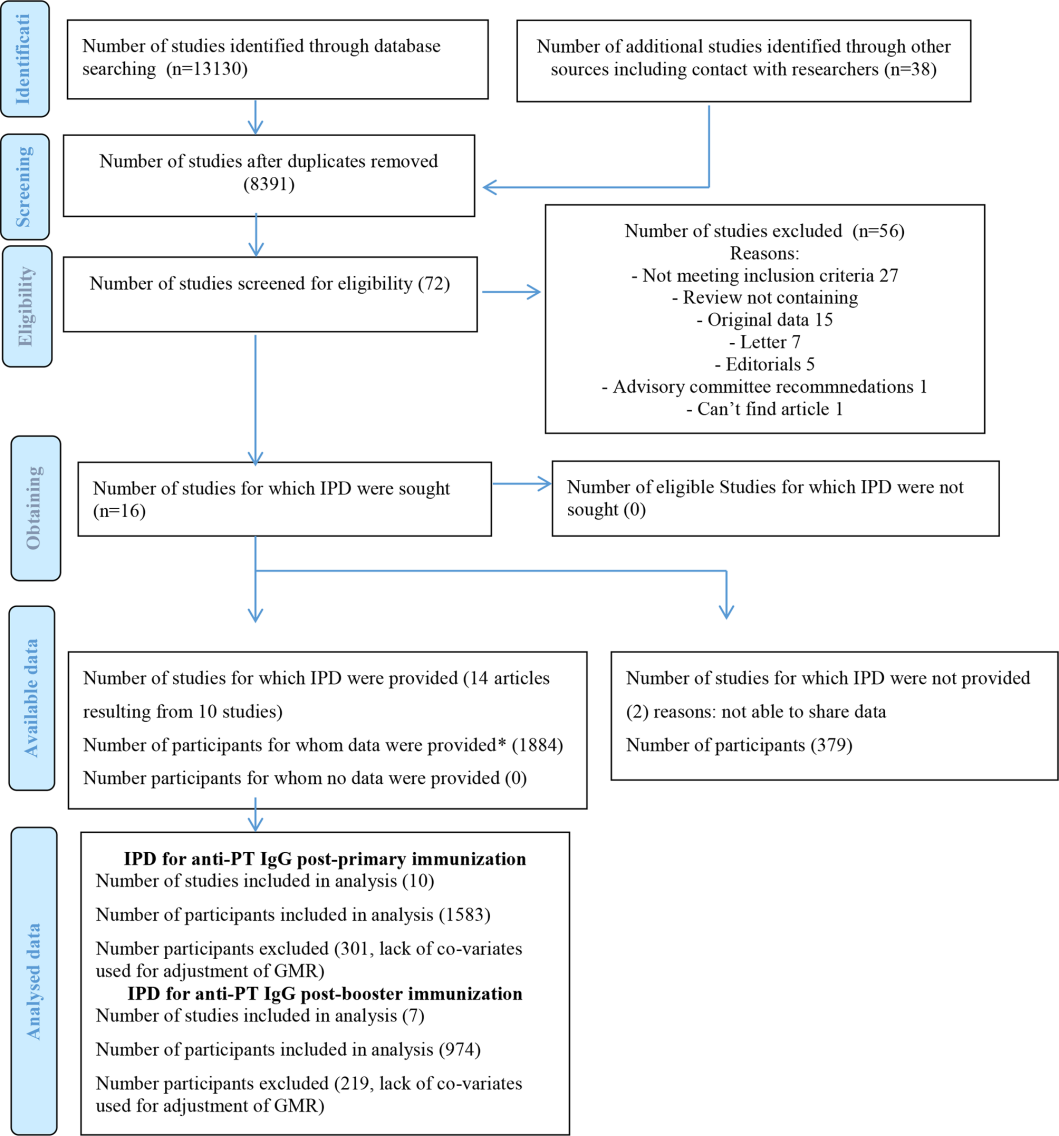
PRISMA IPD Flow Diagram. IPD, individual-participant data; GMR, geometric mean ratio. *In order to confirm data integrity, the main analysis for each specific study was recapitulated and compared to the published data. Adapted from: "http://www.prisma-statement.org/Extensions/IndividualPatientData".

A total of 8391 articles were screened and 72 full-text articles were assessed for eligibility. Sixteen articles met the inclusion criteria, which originated from 12 studies (some studies that included post-primary and post-booster immunization assessment were published in separate and sequential articles leading to higher number of published articles than the number of original articles) (**Figure 1**). All but one of the studies were done in high-income countries and 1 study in a middle-income country (**Table 1**). Women who did not receive Tdap in pregnancy were unimmunized or given placebo in 10/12 studies (8, 14, 15, 21–23, 25, 26, 28–32), given TT in 1/12 study (24, 27) and dT in 1/12 study (16). Infants were immunized with different DTaP vaccines and immunization against Hib was part of DTaP formulations and conjugated to TT in all studies (**Table 1**). Overall, risk of bias was low (**Supplementary Tables 2** and **3**).

**Table 1:** Characteristics of studies identified through the systematic review.

Author [study location, time period(s)]	Study design	Vaccine in pregnancy (timing of immunization in pregnancy)	Vaccines administered to infants for primary immunization and schedule****	Vaccines administered to infants for booster immunization and schedule****	Infants’ outcomes measure (antibody levels to vaccine specific antigens)
Barug (Netherlands, 2014-2016) (21)*	Randomized controlled trial	Boostrix, GSK (30-32 WG).	Infanrix Hexa, GSK; Synflorix, GSK; at 3, 5 months of age	Infanrix Hexa, GSK; Synflorix, GSK; at 11 months of age	PT, FHA, PRN
Barug (Netherlands, 2014-2016) (22)*	Randomized controlled trial	Boostrix, GSK (30-32 WG).	Infanrix Hexa, GSK; Synflorix, GSK; at 3, 5 months of age	Infanrix Hexa, GSK; Synflorix, GSK; at 11 months of age	DT, TT, Hib, SPN 1, 4, 5, 6B, 7F, 9V, 14, 18C, 19F, 23F, 6A, 19A
Halperin (Canada, 2007-2011 and 2012-2014) (16)*	Randomized controlled rial	Adacel, Sanofi Pasteur (33–35 WG)	DTaP-IPV-Hib; Pediacel, Sanofi Pasteur; 2, 4, and 6, months	DTaP-IPV-Hib; Pediacel, Sanofi Pasteur; 12 months	PT, FHA,PRN, FIM2/3, TT, DT, Hib
Hardy-Fairbanks (US, 2006, 2008-2009) (23)	Retrospective cohort study	Adacel, Sanofi Pasteur (any trimester [Trimester 1: 4 women Trimester 2: 8 women Trimester 3: 4 women])	Tdap group: Pediarix, GSK; 2, 4, 6 months Control group: Pediarix, GSK or Pentacel, Sanofi Pasteur or Infanrix, GSK or a combination of these vaccines; 2, 4, 6 months	Tdap group: Infanrix, GSK or TriHIBit, Sanofi Pasteur or Pediarix^®^, GSK; 12-18 months. Control group: Infanrix, GSK or Pediarix^®^, GSK or Daptacel^®^ Sanofi Pasteur, or Pentacel^®^, Sanofi Pasteur; 12-18 months	PT, FHA, PRN, FIM2/3, TT, DT, HBV, Polio1/2/3
Hoang (Vietnam, 2013-2013) (24)*	Randomized controlled rial	Adacel, Sanofi Pasteur (18-36 WG)	Infanrix Hexa, GSK Biologicals; 2, 3, 4 months of age	N/A	TT, DT, PT, FHA, PRN
Klein (US, 2014-2015) (25)*	Randomized controlled trial***	Pertussis vaccine (trade name N/Av), (timing, N/Av)	Infanrix Hexa, GSK Pentacel, Sanofi Pediarix, GSK co-administered with with PCV13 (Prevnar 13, Pfizer Inc.) at 2, 4, 6 months	Hiberix, GSK ActHIB, Sanofi Pentacel, Sanofi at 11 months	PT, FHA, PRN, TT, DT, Hib, HBV, Polio (types 1, 2, 3)
Ladhani (UK, 2012-2014) (26)	Case-control study with historical cohort	Repevax^®^, Sanofi Pasteur (median interval between immunization and delivery: 9.9 WG)	Pediacel, Sanofi Pasteur; 2, 3, 4 months Prevenar-13^®^, Pfizer; 2, 4 months Neivac-C, Pfizer or Menjugate, Sanofi Pasteur or Meningitec, Pfizer; 3, 4 months	N/A	PT, FHA, FIM2/3, TT, DT, Hib, MenC, SPN 1, 3, 4, 6A, 6B, 7B, 9V, 14, 18C, 19A, 19F, 23F.
Maertens (Belgium, 2012-2014) (14)*	Prospective controlled cohort study	Boostrix, GSK (22-33 WG)	Infanrix Hexa^®^, GSK; 8, 12 and 16 weeks of age	N/A	TT, DT, PT, FHA, PRN
Maertens (Belgium, 2012-2014) (15)*	Prospective controlled cohort study	Boostrix, GSK (22-33 WG)	Infanrix Hexa^®^, GSK; 8, 12 and 16 weeks of age	Infanrix Hexa^®^, GSK Biologicals; 15 months of age (booster immunization)	TT, DT, PT, FHA, PRN
Maertens (Vietnam, 2013-2013) (27)*	Randomized controlled rial	Adacel, Sanofi Pasteur (18-36 WG)	Infanrix Hexa, GSK Biologicals; 2, 3, 4 months of age	Infanrix Hexa^®^, GSK; Second year of life (mean age Tdap group: 22.18 months; mean age control group: 21.44 months)	TT, DT, PT, FHA, PRN
Maertens (Belgium, 2011-2015) (28)*	Prospective controlled cohort study	Boostrix, GSK (22-33 WG)	Prevenar-13, Pfizer; 2, 4 months Infanrix Hexa, GSK; 2, 3, 4 months	Prevenar-13, Pfizer at 12 months	SPN 1, 3, 4, 6A, 6B, 7B, 9V, 14, 18C, 19A, 19F, 23F.
Orije et al. (Belgium, 2015-2019) (29)*	Prospective controlled cohort study	Boostrix, GSK (Mean GA at immunization: 29.3 weeks (13.4-36.9 weeks).	Hexyon, Sanofi Pasteur at 8-12-16 weeks. Synflorix, GSK at 8-16 weeks and 12 months.	Hexyon, Sanofi Pasteur at 15 months. Synflorix, GSK at 12 months. Neivac-C, Pfizer at 15 months.	PT, FHA, PRN, TT, DT, Hib, HBV, Polio (types 1, 2, 3)
Munoz (US, 2008-2012) (8)*	Randomized controlled rial	Adacel, Sanofi Pasteur (30-32 WG)	Pentacel, Sanofi Pasteur; 2, 4, 6 months	Pentacel^®^, Sanofi Pasteur; 12 months	PT, FHA, PRN, FIM2/3, TT, DT
Perret (Australia, Canada, Czech Republic, Finland, Italy and Spain, 2016-2018) (30)*	Phase IV, multi-center, observer-blind, randomized, placebo-controlled	Boostrix, GSK (27–36 WG)	2 or 3 doses of DTaP-HepB-IPV/Hib (Infanrix Hexa, GSK) co-administered with PCV13 (Prevnar 13, Pfizer Inc.) at 2 and 4 months; or 3 and 5 months; or 2, 4 and 6 months; or 2, 3 and 4 months of age, according to the different countries’ routine primary immunization schedules	N/A	PT, FHA, PRN, TT, DT, Hib, HBV, SPN 1, 3, 4, 6A, 6B, 7B, 9V, 14, 18C, 19A, 19F, 23F; Polio (types 1, 2, 3),
Rice (UK, 2014-2016) (31)*	Prospective controlled cohort study	Repevax, Sanofi Pasteur (prior to July 2014) and Boostrix-IPV GSK (after July 2014) (N/Av).	DtaP5-IPV-Hib Pediacel, Sanofi Pasteur or DtaP3-IPV-Hib (Infanrix-IPV-Hib; GSK) at 2, 3 and 4 months of age. Prevenar 13 (Pfizer) at 2 and 4 months of age	N/A	PT, FHA, PRN, TT, DT, Hib, SPN 1, 3, 4, 6A, 6B, 7B, 9V, 14, 18C, 19A, 19F, 23F.
Zimmermann (Australia, 2013-2016) (32)*	Randomized controlled trial**	Boostrix, GSK (N/Av)	Infanrix Hexa, GSK; Prevenar 13, Wyeth; At 6 weeks, 4 months and 6 months of age	Menitorix, GSK; at 12 months of age	PT, FHA, PRN, TT, DT, Hib, SPN 1, 3, 4, 6A, 6B, 7B, 9V, 14, 18C, 19A, 19F, 23F. Polio (types 1, 2, 3), MenC, measles, mumps and rubella

*Included in the individual-participant data meta-analysis.

**The original study randomized infants to receive or not Bacillus Calmette–Guérin at birth. For the purpose of this meta-analysis, women were not randomized to receive Tdap or not in pregnancy.

***The original study randomized infants to receive different immunization schedules. For the purpose of this meta-analysis, women were not randomized to receive Tdap or not in pregnancy.

****Vaccination schedule per published articles and their composition is:

Boostrix: TT (5 Lf), DT (2.5 Lf), PT (8 mcg), FHA (8 mcg), PRN (2.5 mcg), Adacel: TT (5 Lf). DT (2 Lf), PT (2.5 mcg), FHA (5 mcg), PRN (3 mcg), FIM2/3 (5 mcg). Repevax: TT (≥20 IU), DT (≥2 IU), PT (2.5 mcg), FHA (5 mcg), PRN (3 mcg), FIM2/3 (5 mcg), polio virus type 1 (40 D antigen units), polio virus type 2 (8 D antigen units), polio virus type 3 (32 D antigen units). Infanrix Hexa: TT (10 Lf), DT (25 Lf), PT (25 mcg), FHA (25 mcg), PRN (8 mcg), HBsAg (10 mcg), polio virus type 1 (40 D antigen units), polio virus type 2 (8 D antigen units), polio virus type 3 (32 D antigen units), Hib (10 mcg). Pediacel: TT (5 Lf), DT (15 Lf), PT (20 mcg), FHA (20 mcg), PRN (3 mcg), FIM2/3 (5 mcg), polio virus type 1 (40 D antigen units), polio virus type 2 (8 D antigen units), polio virus type 3 (32 D antigen units), Pediarix: TT (10 Lf), DT (25 Lf), PT (25 mcg), FHA (25 mcg), PRN (8 mcg), polio virus type 1 (40 D antigen units), polio virus type 2 (8 D antigen units), polio virus type 3 (32 D antigen units), HBsAg (10 mcg). Pentacel: TT (5 Lf), DT (15 Lf), PT (20 mcg), FHA (20 mcg), PRN (3 mcg), FIM2/3 (5 mcg), polio virus type 1 (40 D antigen units), polio virus type 2 (8 D antigen units), polio virus type 3 (32 D antigen units), Hib (10 mcg). Hexyon: TT (≥40 IU), DT (≥20 IU), PT (25 mcg), FHA (25 mcg), polio virus type 1 (40 D antigen units), polio virus type 2 (8 D antigen units), polio virus type 3 (32 D antigen units), HBsAg (10 mcg), Hib (12 mcg). Daptacel: TT (5 Lf), DT (15 Lf), PT (10 mcg), FHA (5 mcg), PRN (3 mcg), FIM2/3 (5 mcg). Hiberix: Hib (10 mcg). ActHIB: Hib (10 mcg). US, United States; Tdap, tetanus-diphtheria-acellular-pertussis; GSK, GlaxoSmithKline; PT, pertussis toxin; FHA, filamentous hemagglutinin; PRN, pertactin; FIM2/3, fimbriae 2/3; TT, tetanus toxoid; DT, diphtheria toxoid; HBV, Hepatitis B virus; UK, United Kingdom; WG, weeks gestation; Hib, Haemophilus influenzae b; MenC, meningococcal C; SPN, Streptococcus pneumoniae; N/Av, not available; N/A, not applicable. IPV, inactivated polio virus; IU, International Unit; HBsAg,hepatitis B surface antigen.

Data from 14/16 articles (10 studies) were received and included in the meta-analysis (**Table 1**). Individual-participant data integrity was confirmed for all studies.

**Figure 2:**
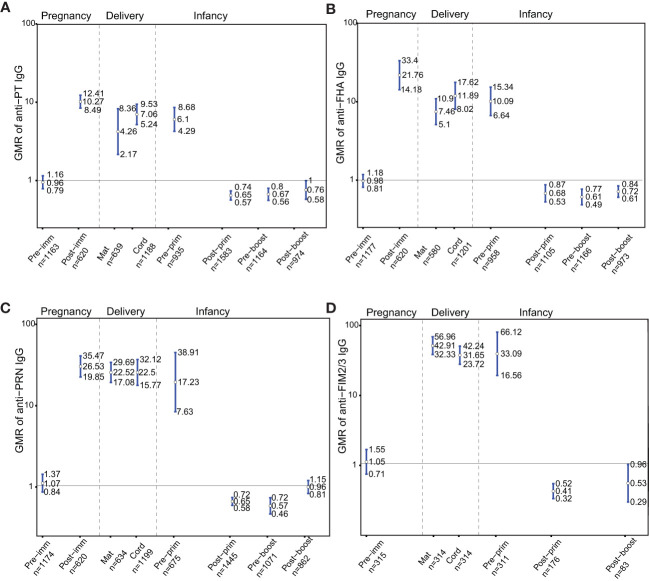
Antibody responses to pertussis antigens. Geometric mean ratio (GMR) of **(A)** anti-pertussis toxin (PT). **(B)** anti-filamentous haemagglutinin (FHA), **(C)** anti-pertactin (PRN), and **(D)** anti-fimbria 2/3 (FIM2/3) IgG levels in women immunized with tetanus-diphtheria-acellular-pertussis (Tdap) *versus* women who did not receive Tdap in pregnancy pre-immunization, post-immunization and at delivery; in infants born to women immunized with Tdap *versus* infants of women not immunized with Tdap in pregnancy pre-primary, post-primary, pre-booster and post-booster immunization with diphtheria-tetanus-acellular-pertussis. For FIM2/3, GMR was not computed post-immunization in pregnancy and pre-booster immunization in infancy, as data were available for one study on these time points precluding meta-analysis. Vertical blue lines indicate the GMR with the 95% confidence interval. Horizontal black line indicates a GMR of 1. The numbers available for meta-analysis are indicated (n). GMR results displayed are derived from mixed-effects models. Pre-imm, pre-immunization; Post-imm, post-immunization; Mat, maternal; Pre-prim, pre-primary; Post-prim, post-primary; Pre-boost, pre-booster; Post-boost, post-booster.

IgG levels against PT, FHA, PRN and FIM2/3 were comparable pre-immunization in women who later received Tdap in pregnancy compared to women who later did not receive Tdap, (GMR 0.96; 95%CI 0.79-1.16), (GMR 0.98; 95%CI 0.81-1.18), (GMR 1.07; 95%CI 0.84-1.37) and (GMR 1.05; 95%CI 0.71-1.55), respectively (**Figures 2A–D**). Anti-PT, anti-FHA, and anti-PRN IgG levels were significantly higher in women who received Tdap in pregnancy post-immunization compared to women who did not receive Tdap, (GMR 10.27; 95%CI 8.49-12.41), (GMR 21.76; 95%CI 14.18-33.4), and (GMR 26.53; 95%CI 19.85-35.47), respectively (**Figures 2A–C**). At delivery, IgG levels against PT, FHA, PRN and FIM2/3 were significantly higher in women who received Tdap in pregnancy compared to women who did not receive Tdap both in maternal and cord sera (**Figures 2A–D**). These higher levels were still maintained pre-primary immunization for anti-PT, anti-FHA, anti-PRN and anti-FIM 2/3 IgG, (GMR 6.1; 95%CI 4.29-8.68), (GMR 10.09; 95%CI 6.64-15.34), (GMR 17.23; 95%CI 7.63-38.91) and (GMR 33.09; 95%CI 16.56-66.12), respectively (**Figures 2A–D**). Infants of women immunized with Tdap in pregnancy had significantly lower anti-PT IgG levels compared with infants of women who did not receive Tdap in pregnancy post-primary and pre-booster immunization with DTaP, (GMR 0.65; 95%CI 0.57-0.74), and (GMR 0.67; 95% CI 0.56-0.8), respectively (**Figure 2A**). A trend was noted post-booster immunization (GMR 0.76; 0.58-1.00) (**Figure 2A**). Infants of women immunized with Tdap in pregnancy had significantly lower anti-FHA IgG levels compared with infants of women who did not receive Tdap in pregnancy post-primary, pre-booster and post-booster immunization with DTaP, (GMR 0.68; 95%CI 0.53-0.87), (GMR 0.61; 95% CI 0.49-0.77), and (GMR 0.72; 95% CI 0.61-0.84), respectively (**Figure 2B**). Infants of women immunized with Tdap in pregnancy had significantly lower anti-PRN IgG levels compared with infants of women who did not receive Tdap in pregnancy post-primary and pre-booster immunization with DTaP, (GMR 0.65; 95%CI 0.58-0.72), and (GMR 0.57; 95% CI 0.46-0.72), respectively (**Figure 2C**). Infants of women immunized with Tdap in pregnancy had significantly lower anti- FIM2/3 IgG levels compared with infants of women who did not receive Tdap in pregnancy post-primary and post-booster immunization with DTaP, (GMR 0.41; 95%CI 0.32-0.52), and (GMR 0.53; 95% CI 0.29-0.96), respectively (**Figure 2D**).

The GMRs of IgG against PT, FHA and PRN pre-immunization in pregnancy and at delivery in women immunized in pregnancy *vs.* unimmunized women and their infants stratified by type of vaccine administered in pregnancy (Adacel *versus* Boostrix) were also computed (**Table 2**).

**Table 2:** Geometric mean ratio of anti-*Bordetella pertussis* IgG levels in women immunized with tetanus-diphtheria-acellular-pertussis (Tdap) *versus* women who did not receive Tdap in pregnancy and their infants stratified by type of vaccine administered in pregnancy.

	Geometric mean ratio (95% CI, n)		
	Pertussis toxin	Filamentous hemagglutinin	Pertactin
**Adacel in pregnancy**			
Pregnancy*			
Pre-immunization	1.17 (0.94-1.45, 409)	1.07 (0.83-1.38, 419)	0.96 (0.58-1.61, 417)
**Delivery**			
Maternal	5.16 (2.93-9.1, 411)	10.11 (7.54-13.56, 404)	22.91 (16.66-31.51, 403)
Cord	4.47 (2.91-6.86, 386)	6.57 (3.3-13.1, 395)	18.32 (9.08-36.96, 398)
Infancy**			
Pre-primary	4.14 (2.95-5.82, 341)	6.96 (2.38-20.36, 361)	12.49 (5.84-26.68, 361)
Post-primary	0.83 (0.68-1, 338)	0.69 (0.47-1, 338)	0.66 (0.53-0.82, 334)
Post-booster	0.76 (0.62-0.94, 342)	0.75 (0.59-0.96, 343)	1.11 (0.9-1.37, 346)
**Boostrix in pregnancy**			
Pregnancy*			
Pre-immunization	0.86 (0.64-1.15, 723)	0.9 (0.7-1.16, 727)	1.01 (0.81-1.27, 726)
**Delivery**			
Maternal	2.75 (0.27-27.89, 197)	4.07 (0.42-39.16, 145)	12.42 (2.54-60.71, 200)
Cord	8.49 (6.81-10.57, 771)	14.31 (8.96-22.85, 775)	20.67 (14.2-30.09, 772)
**Infancy**			
Pre-primary	7.01 (4.42-11.13, 563)	11.68 (7.46-18.28, 566)	17.45 (11.94-25.52, 616)
Post-primary	0.58 (0.51-0.66, 843)	0.67 (0.42-1.07, 365)	0.6 (0.51-0.7, 734)
Pre-booster	0.59 (0.5-0.69, 576)	0.61 (0.41-0.9, 586)	0.47 (0.35-0.63, 481)
Post-booster	0.78 (0.37-1.65, 288)	0.77 (0.49-1.21, 286)	0.8 (0.52-1.22, 174)

Given that Fimbria antigens exists only in Adacel, separate analyses were not performed as the analyses presented in **Figure 2D** were restricted to women who received Adacel and their infants.

*Post-immunization in pregnancy analyses were not performed, as model fitting was not feasible due to the small number of studies eligible for inclusion.

**Pre-booster immunization in infancy analyses were not performed, as model fitting was not feasible due to the small number of studies eligible for inclusion.

**Figure 3:**
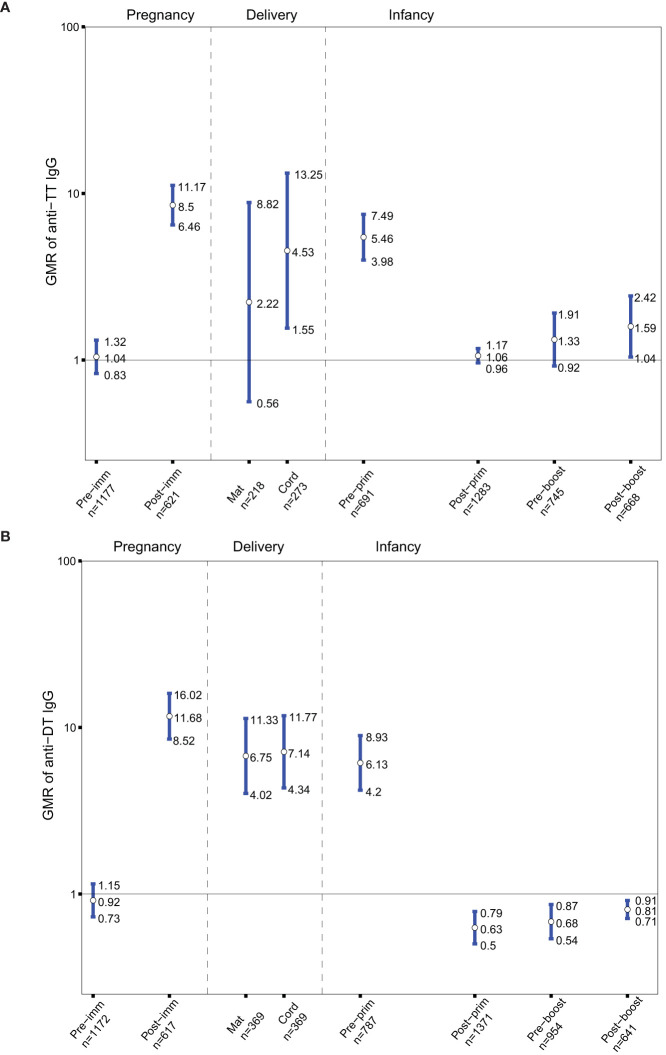
**(A)** Antibody responses to tetanus-toxoid (TT). Geometric mean ratio (GMR) of anti-TT IgG levels in women immunized with tetanus-diphtheria-acellular-pertussis (Tdap) *versus* women who did not receive Tdap or diphtheria and tetanus toxoids (dT) or TT vaccine in pregnancy pre-immunization, post-immunization and at delivery; in infants born to women immunized Tdap *versus* infants of women not immunized with Tdap dT, or TT vaccine in pregnancy pre-primary, post-primary, pre-booster and post-booster immunization with diphtheria-tetanus-acellular-pertussis vaccine. **(B)** Antibody responses to diphtheria-toxoid (DT). GMR of anti-DT IgG levels in women immunized with Tdap *versus* women who did not receive Tdap or dT vaccine in pregnancy pre-immunization, post-immunization and at delivery; in infants born to women immunized Tdap *versus* infants of women not immunized with Tdap or dT vaccine in pregnancy pre-primary, post-primary, pre-booster and post-booster immunization with diphtheria-tetanus-acellular-pertussis vaccine. Vertical blue lines indicate the GMR with the 95% confidence interval. Horizontal black line indicates a GMR of 1. The numbers available for meta-analysis are indicated (n). GMR results displayed are derived from mixed-effects models. Pre-imm, pre-immunization; Post-imm, post-immunization; Mat, maternal; Pre-prim, pre-primary; Post-prim, post-primary; Pre-boost, pre-booster; Post-boost, post-booster.

As Tdap vaccines administered in pregnancy also include tetanus antigens, the GMRs of anti-TT IgG in the groups of women immunized with Tdap *vs.* unimmunized in pregnancy and their infants before and after receipt of DTaP in infancy were determined. Anti-TT IgG levels were comparable pre-immunization in women who later received Tdap in pregnancy compared to women who later did not receive Tdap, TT or dT vaccines in pregnancy (**Figure 3A**). Anti-TT IgG levels were higher in women who received Tdap in pregnancy post-immunization compared to women who did not receive Tdap, TT or dT vaccines in pregnancy (**Figure 3A**).Anti-TT IgG levels were higher at birth, pre-primary immunization and post-booster immunization with DTaP, in infants born to women immunized with Tdap in pregnancy compared with infants of women who did not receive Tdap, TT or dT vaccines in pregnancy, (GMR 4.53; 95%CI 1.55-13.52), (GMR 5.46; 95% CI 3.98-7.49), and (GMR 1.59; 95% CI 1.04-2.42), respectively (**Figure 3A**).

Tdap vaccines administered in pregnancy also include diphtheria antigens, thus the GMRs of anti-DT IgG in the groups of women immunized with Tdap *vs.* unimmunized in pregnancy and their infants before and after receipt of DTaP in infancy were also sought. Anti-DT IgG levels were comparable pre-immunization in women who later received Tdap in pregnancy compared to women who later did not receive Tdap or dT (**Figure 3B**). Anti-DT IgG levels were higher in women who received Tdap in pregnancy post-immunization and at delivery compared to women who did not receive Tdap or dT. Anti-DT IgG levels were higher in infants born to Tdap-immunized women at birth and pre-primary immunization (**Figure 3B**). Infants of women immunized with Tdap in pregnancy had significantly lower anti-DT IgG levels compared with infants of women who did not receive Tdap or dT in pregnancy post-primary, pre-booster and post-booster immunization with DTaP, (GMR 0.63; 95%CI 0.5-0.79), (GMR 0.68; 95% CI 0.54-0.87), and (GMR 0.81; 95% CI 0.71-0.91), respectively (**Figure 3B**).

**Figure 4:**
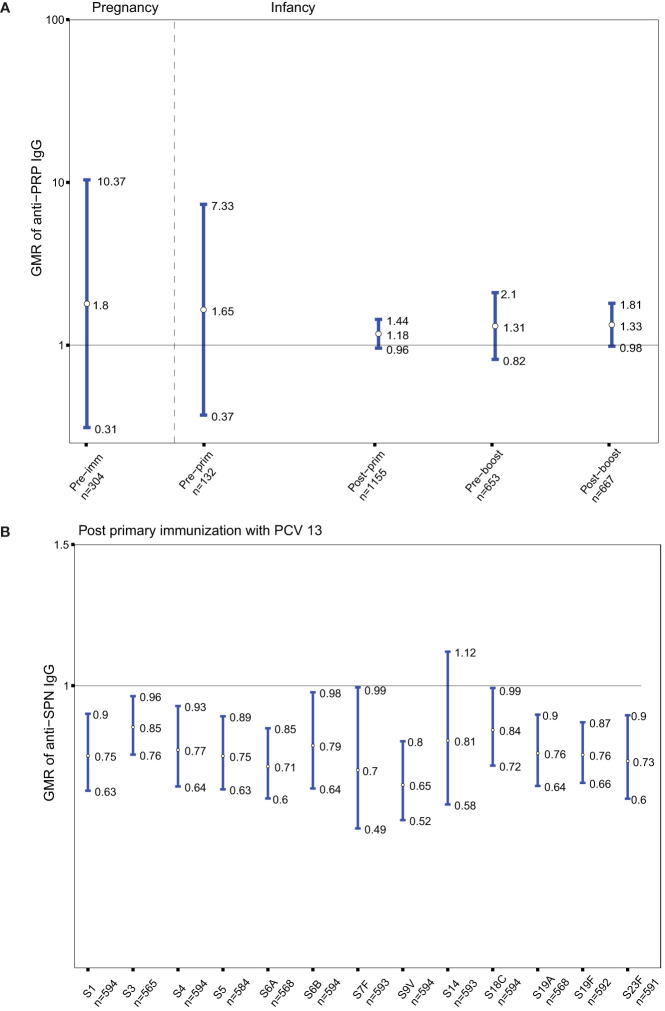
**(A)** Antibody responses to *Haemophilus influenza* type b (polyribosylribitol phosphate [PRP]). Geometric mean ratio (GMR) of anti-PRP IgG levels in women immunized with tetanus-diphtheria-acellular-pertussis (Tdap) *versus* women who did not receive Tdap or diphtheria and tetanus toxoids (dT) or TT vaccine in pregnancy pre-immunization; in infants born to women immunized Tdap *versus* infants of women not immunized with Tdap, dT, or TT vaccine in pregnancy pre-primary, post-primary, pre-booster and post-booster immunization with diphtheria-tetanus-acellular-pertussis-Hib vaccine. **(B)** Antibody responses to *Streptococcus pneumoniae* (SPN). GMR of anti-SPN IgG levels in infants born to women immunized with Tdap compared with infants of women who did not receive Tdap or dT vaccine in pregnancy after their primary immunization with pneumococcal conjugate vaccine 13 (PCV 13). Vertical blue lines indicate the GMR with the 95% confidence interval. Horizontal black line indicates a GMR of 1. The numbers available for meta-analysis are indicated (n). GMR results displayed are derived from mixed-effects models. Pre-imm, pre-immunization; Pre-prim, pre-primary; Post-prim, post-primary; Pre-boost, pre-booster; Post-boost, post-booster; S, Serotype.

As Tdap vaccines administered in pregnancy include tetanus antigens, the GMRs of anti-PRP IgG in the groups of women immunized with Tdap *vs.* unimmunized in pregnancy and their infants before and after receipt of Hib vaccines conjugated to TT were determined. Anti-PRP IgG levels were not significantly different in infants born to women immunized with Tdap in pregnancy compared with infants of women who did not receive Tdap, TT or dT vaccines in pregnancy at all-time points (**Figure 4A**).

Given that Tdap vaccines administered in pregnancy include diphtheria antigens, the GMRs of anti-SPN IgG in the groups of infants born to women immunized with Tdap *vs.* unimmunized in pregnancy after receipt of pneumococcal vaccines conjugated to DT were also computed. Anti-SPN IgG levels were significantly lower in infants born to women immunized with Tdap in pregnancy post-primary immunization with PCV13 compared with infants of women who did not receive Tdap or dT vaccines in pregnancy for serotypes 1, 3, 4, 5, 6A, 6B, 7F, 9V, 18C, 19A, 19F, 23F with a reduction ranged from 15 to 35% (**Figure 4B**).

**Figure 5:**
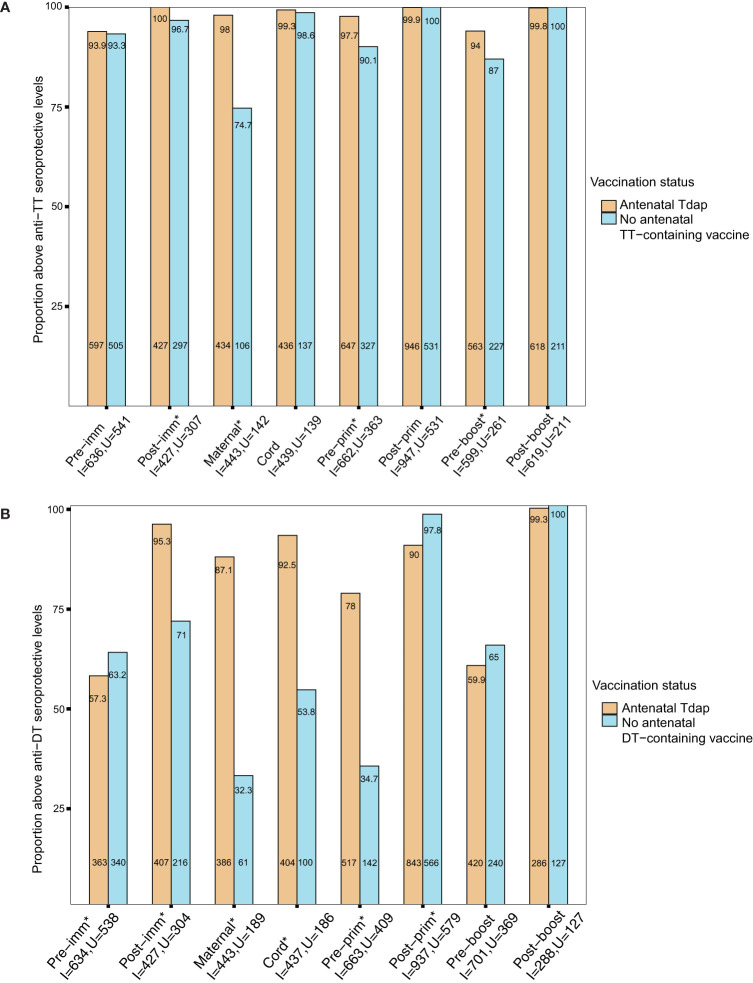
**(A)** Seroprotection rates against tetanus diseases. Rates of participants with anti–tetanus toxoid (TT) IgG ≥0.1 IU/mL in women immunized with tetanus-diphtheria-acellular pertussis (Tdap) *versus* women who did not receive Tdap, diphtheria and tetanus toxoids (dT), or TT vaccine in pregnancy pre-immunization, post-immunization and at delivery; in infants born to women immunized Tdap *versus* infants of women not immunized with Tdap, dT or TT vaccine in pregnancy pre-primary, post-primary, pre-booster and post-booster immunization with diphtheria-tetanus-acellular pertussis vaccine (*comparisons with p-values <0.05). P-values: Pre-imm: p=0.806; **Post-imm: p = 0.001**; **Maternal: p<0.001**; Cord: p = 0.754; **Pre-prim: p<0.001**;Post-prim: p =1; **Pre-boost: p=0.001**;Post-boost: p =1. **(B)** Seroprotection rates against diphtheria diseases. Rates of participants with anti-DT IgG ≥0.1 IU/mL in women immunized with tetanus-diphtheria-acellular pertussis (Tdap) *versus* women who did not receive Tdap or dT vaccine in pregnancy pre-immunization, post-immunization and at delivery; in infants born to women immunized Tdap *versus* infants of women not immunized with Tdap or dT vaccine in pregnancy pre-primary, post-primary, pre-booster and post-booster immunization with diphtheria-tetanus-acellular pertussis vaccine (*comparisons with p-values < 0.05). P-values: **Pre-imm: p=0.045**; **Post-imm: p<0.001**; **Maternal: p<0.001**; **Cord: p<0.001**; **Pre-prim: p<0.001**; **Post-prim: p<0.001**; Pre-boost: p=0.116; Post-boost: p=0.863. Seroprotection rates were compared using the chi-squared test. Pre-imm, pre-immunization; Post-imm, post-immunization; Pre-prim, pre-primary; Post-prim, post-primary; Pre-boost, pre-booster; Post-boos, post-booster; I, immunized; U, Unimmunized. Absolute numbers are shown in the bottom and percentages in the top of the bars.

In order to provide insights whether the changes in antigen-specific GMRs following Tdap immunization in pregnancy affect protection from infections in women and their infants, seroprotection rates against diseases for which there are correlates of protection (tetanus, diphtheria, Hib, IPD) were explored. Nearly 93% of women had seroprotective antibody levels against tetanus disease pre-immunization (**Figure 5A**). This rate increased to nearly 100% at birth in women immunized with Tdap during pregnancy and in cord of pregnant women immunized and unimmunized with Tdap, TT or dT vaccines in pregnancy (**Figure 5A**). Infants of women immunized with Tdap in pregnancy had significantly higher seroprotection rates against tetanus pre-primary and pre-booster immunization compared with infants of women unimmunized with Tdap, TT or dT vaccines in pregnancy, 98% (647/662) *vs* 90.1% (327/363), and 92% (563/599) *vs* 87% (227/261), p<0.001, p=0.001, respectively (**Figure 5A**). Infants of women immunized with Tdap in pregnancy had comparable high seroprotection rate post-primary and post-booster immunization compared with infants of women unimmunized with Tdap, TT or dT vaccines in pregnancy, 99% (946/947) *vs* 100% (531/531), and 99% (618/619) *vs* 100% (211/211), respectively, p=1 for both comparisons (**Figure 5A**).

In addition, the seroprotection rates against diphtheria disease in the groups of women immunized with Tdap *vs.* unimmunized in pregnancy and their infants before and after receipt of DTaP in infancy were also determined. Nearly 60% of pregnant women had seroprotection antibody levels against diphtheria disease pre-immunization (**Figure 5B**). Seroprotection rates against diphtheria disease were significantly higher in women immunized with Tdap in pregnancy post-immunization and at birth compared with women who did not receive Tdap or dT vaccine in pregnancy (**Figure 5B**). Infants of women immunized with Tdap in pregnancy had significantly higher seroprotection rates against diphtheria pre-primary immunization and significantly lower seroprotection rates post-primary immunization compared with infants of women who did not receive Tdap or dT vaccines in pregnancy, 78% (517/663) *vs* 35% (142/409) and 90% (843/937) *vs* 98% (566/579), all p<0.001 (**Figure 5B**). Nearly 60% (420/701) of infants born to women immunized with Tdap in pregnancy had seroprotective anti-DT levels pre-booster immunization, increasing to 99% (286/288) post-booster immunization (**Figure 5B**).

**Figure 6:**
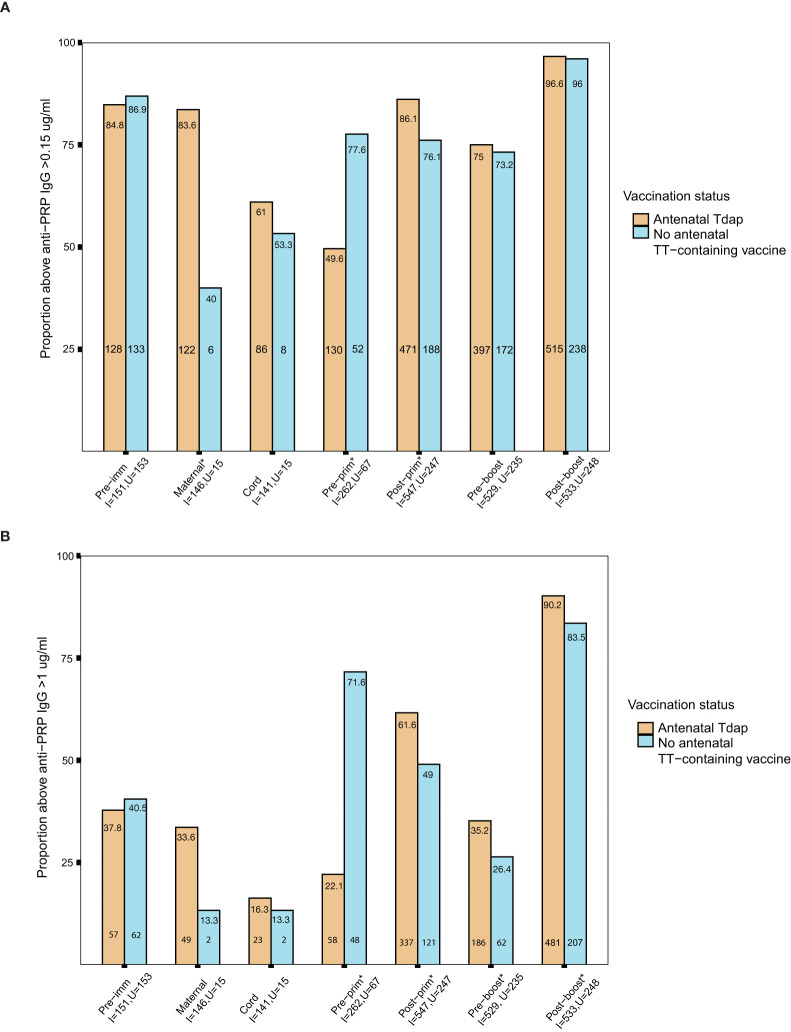
**(A)** Short-term seroprotection rates against *haemophilus influenzae* type b (Hib) disease. Rates of subjects with anti–polyribosylribitol phosphate (PRP) IgG ≥0.15 mcg/ml in women immunized with tetanus-diphtheria-acellular pertussis (Tdap) *versus* women who did not receive Tdap vaccine, diphtheria and tetanus toxoids (dT) or tetanus-toxoid (TT) vaccine in pregnancy pre- immunization and at delivery; in infants born to women immunized Tdap *versus* infants of women not immunized with Tdap, dT or TT vaccine in pregnancy pre-primary, post-primary, pre-booster and post-booster immunization with diphtheria-tetanus-acellular-pertussis-Hib vaccine (*comparisons where p-values <0.05). P-values: Pre-imm: p= 0.707; **Maternal: p<0.001**; Cord: p=0.765; **Pre-prim: p<0.001**; **Post-prim: p= 0.001**; Pre-boost: p= 0.651; Post-boost:p=0.801. **(B)** Long-term seroprotection rates against Hib disease. Rates of subjects with anti–PRP IgG ≥1 mcg/ml in women immunized with Tdap *versus* women who did not receive Tdap, dT or TT vaccine in pregnancy pre- immunization and at delivery; in infants born to women immunized Tdap *versus* infants of women not immunized with Tdap, dT or TT vaccine in pregnancy pre-primary, post-primary, pre-booster and post-booster immunization with diphtheria-tetanus-acellular-pertussis-Hib vaccine (*comparisons where p-values <0.05). P-values: Pre-imm: p= 0.705; Maternal: p= 0.189; Cord: p=1; **Pre-prim: p<0.001**; **Post-prim: p= 0.001**; **Pre-boost: p= 0.021**; **Post-boost: p=0.009**. Seroprotection rates were compared using the chi-squared test. Pre-imm, pre-immunization; Pre-prim, pre-primary; Post-prim, post-primary; Pre-boost, pre-booster; Post-boos, post-booster; I, immunized; U, Unimmunized. Absolute numbers are shown in the bottom and percentages in the top of the bars.

The seroprotection rates against Hib in the groups of women immunized with Tdap *vs.* unimmunized in pregnancy and their infants before and after receipt of Hib vaccines conjugated to TT, were calculated applying both short and long term cut-offs for protection. Nearly 85% of pregnant women had protective antibody levels against Hib using the short term cut off for protection pre-immunization (**Figure 6A**). In addition, infants of women immunized with Tdap in pregnancy compared with infants of women who did not receive Tdap, TT or dT vaccines in pregnancy had significantly higher seroprotection rates against Hib post-primary immunization 86% (471/547) *vs* 76% (188/247), comparable rates pre-booster immunization 75% (397/529) *vs* 73% (172/235), and post-booster immunization 97% (515/533) *vs* 96% (238/248), p=0.001, p=0.651, p=0.801, respectively, using the cut off of short term protection (**Figure 6A**). Applying the cut-off of long-term protection against Hib, infants of women immunized with Tdap in pregnancy had significantly higher seroprotection rates against Hib post-primary immunization 62% (337/547) *vs* 49% (121/247), pre-booster immunization 35% (186/529) *vs* 26% (62/235), and post-booster immunization 90% (481/533) *vs* 83% (207/248), compared with infants of women who did not receive Tdap, TT or dT vaccines in pregnancy, p=0.001, p=0.021, p=0.009, respectively (**Figure 6B**).

**Figure 7:**
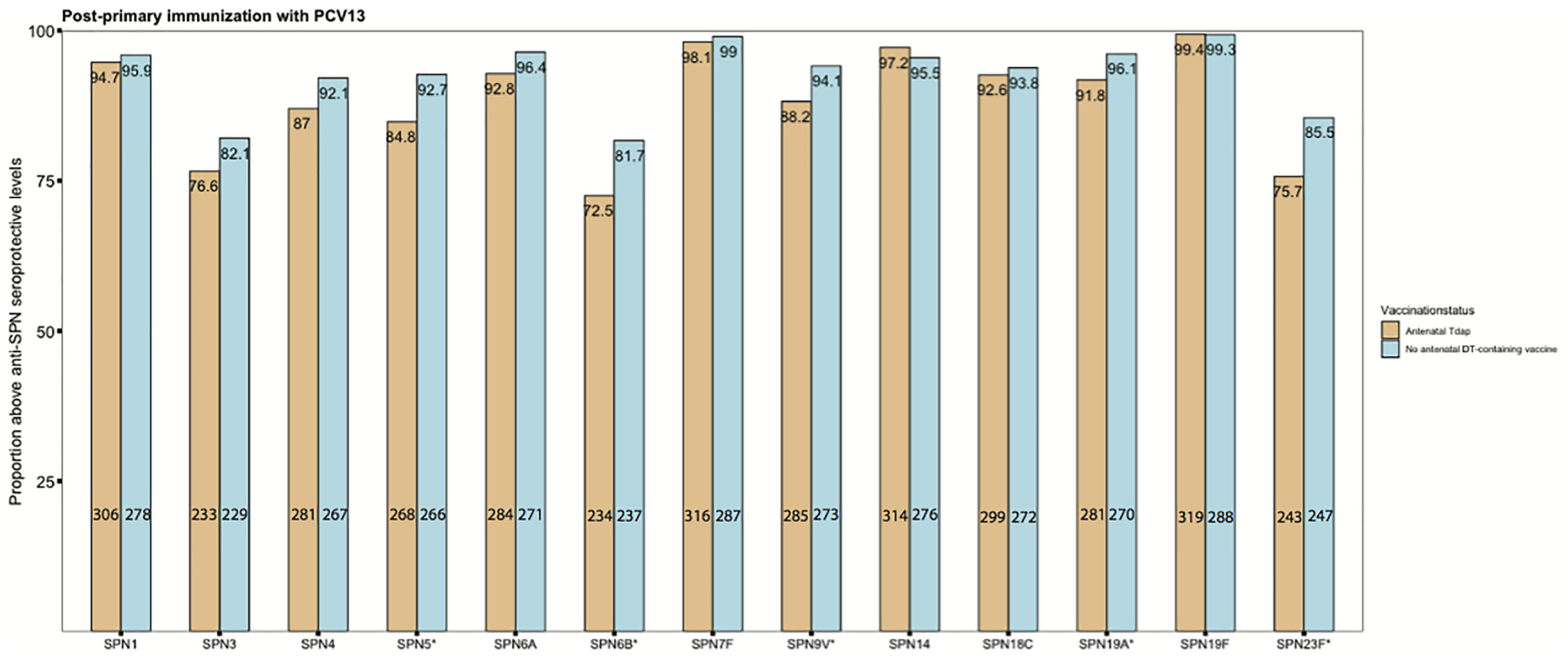
Seroprotection rates against invasive pneumococcal disease. Rates of subjects with anti–*streptococcus pneumonia* (SPN) IgG levels ≥0.35 mg/mL in infants born to women immunized tetanus-diphtheria-acellular-pertussis (Tdap) versus infants of women not immunized with Tdap or diphtheria and tetanus toxoids vaccine in pregnancy after their primary immunization with pneumococcal conjugate vaccine 13 (PCV13) (*comparisons where p-values <0.05). Number of infants born to women Tdap-immunized and unimmunized in pregnancy was in the range of 304-323 and 279-290, respectively, for the different serotypes. P-values for the specific serotypes: SPN1: p=0.642; SPN3: p= 0.13; SPN4: p= 0.057; **SPN5: p= 0.004**; SPN6A: p= 0.079; **SPN6B: p= 0.009**; SPN7F: p= 0.607; **SPN9V: p= 0.016**; SPN14: p= 0.358; SPN18C: p=0.661; **SPN19A: p= 0.048**; SPN19F: p=1; **SPN23F: p=0.003**. Absolute numbers are shown in the bottom and percentages in the top of the bars. Seroprotection rates were compared using the chi-squared test.

Seroprotection rates against SPN following primary immunization with PCV conjugated to DT were also explored. Post-primary immunization with PCV-13, seroprotection rates against 5/13 SPN serotypes were significantly lower in infants of women immunized with Tdap in pregnancy compared with infants of women who did not receive Tdap or dT vaccines in pregnancy, SPN5 85% (268/316) *vs* 93% (266/287), p=0.004; SPN6B 72% (234/323) *vs* 82% (237/290),p=0.009; SPN9V 88% (285/323) *vs* 94% (273/290),p=0.016; SPN19A 92% (281/306) *vs* 96% (270/281), p=0.048; SPN23F 76% (243/321) *vs* 85% (247/289), p=0.003, (**Figure 7**).

## Discussion

This unique, large (n=1583), international and longitudinal mother-infant meta-analysis of individual-participant data from 10 studies has enabled us to clearly quantify the magnitude of immune response to Tdap vaccine in pregnant women and their infants at delivery and pre-primary immunization. We also were able to quantify the reduction in immune responses to pertussis, diphtheria and 12/13 SPN serotypes following primary immunization in infants born to women immunized with Tdap in pregnancy. In addition, we showed that for some antigens this persisted beyond booster infant immunization. Moreover, this reduction in antibody levels resulted in lower seroprotection rates for 5/13 SPN serotypes and for diphtheria after primary immunization. In contrast, enhanced immune responses to tetanus and Hib vaccines conjugated to TT were observed in infants born to women immunized with Tdap in pregnancy. These data have important implications in establishing the effect of Tdap immunization in pregnancy on the antibody immune responses to different vaccine antigens in infancy and inform policy makers in countries where programs of immunization against pertussis in pregnancy have been recommended or being considered.

Studies prior to the implementation of immunization against pertussis in pregnancy suggested that higher pre-existing maternally derived antibody levels could have a suppressive effect on infants’ immune responses to primary immunization against pertussis and other antigens (10–13). This meta-analysis provides further support to these findings in the era of Tdap immunization in pregnancy and also extends these findings to booster immunization. This reduction might potentially put infants born to women immunized against pertussis in pregnancy at increased risk for pertussis disease later in their infancy. However, current surveillance data do not indicate that this reduction in anti-*B. pertussis* antibody levels is clinically relevant. This is because the incidence of pertussis disease in infants after primary and booster immunization did not significantly increase after the introduction of a maternal pertussis immunization program in the UK and was at 2.2 per 100,000 in infants 1-4 years of age (33). In a study conducted in the US between 2010-2015, effectiveness of immunization against pertussis in pregnancy was 66% among infants who received 3 primary immunization doses against pertussis and aged <1 year (34). However, additional disease burden data are needed to definitely assess the true clinical significance of such reduction as the cohort of infants born to pertussis-vaccinated women is increasing (35).

The general mechanism of this modification of immune responses has not been fully investigated. Inhibition of B cell activation through the FcγRIIB on B cells has been proposed. Specifically, vaccine antigen–antibody complexes cross-link the B-cell receptor with the FcγRIIB, thus inhibiting antigen specific B-cell activation (36). It was recently shown in an influenza mice model that inhibition of immune response to immunization following influenza immunization in pregnancy was antigen-specific and correlated with maternal antibodies in a dose-dependent manner and was associated with reduction in the number of germinal center B cells that differentiate into plasma cells and memory B cells (37). This might potentially explain the durable effect of immunization in pregnancy on booster immunization in infancy. Inhibition of B cell *via* epitope masking is another suggested mechanism. In this mechanism, the B cell epitopes on a vaccine antigen(s) are covered by antibodies and thus are not recognized by B cells (38). However, this does not explain the inhibitory effect observed following booster immunization.

As current formulations of Tdap vaccines used in pregnancy also include tetanus toxoids and diphtheria toxoids, infants’ immune responses to TT and DT components of vaccines and vaccines conjugated these toxoids as carrier proteins (e.g. Hib vaccine, and PCVs) might also be modified. In this large meta-analysis, we were able to accurately quantify the reduced immune responses to diphtheria and some SPN serotypes in infants born to women immunized with Tdap in pregnancy after primary and booster immunization, and to show lower seroprotection rates for diphtheria and some SPN serotypes after primary immunization. This might increase the risk of infection with these pathogens in infants born to women immunized with Tdap in pregnancy. Although diphtheria disease incidence has decreased since the implementation of 3 doses of DT-containing vaccines, outbreaks do still occur, especially in low-middle income countries and among unimmunized subjects (39). In high-income countries, diphtheria is a rare disease (40). To the best of our knowledge, the clinical significance of the reduction of seroprotection rates against SPN has not yet been published. While anti-SPN >0.35 ug/ml cut-off is used for licensure of pneumococcal vaccines, some studies showed that SPN correlates of protection are serotype-specific, and are different for protection against disease *versus* colonization, which further complicates the interpretation of immunogenicity data we present in this study (41, 42). In addition, there have been changes in the pneumococcal immunization programs in different countries and these should be considered in the setting of immunization with Tdap in pregnancy. For example, in the UK, PCV primary immunization has been reduced from 2 primary doses followed by a booster dose to a one primary dose followed by a booster dose (43).

The association between Tdap administration in pregnancy and reduced immune response to immunization with PCV13 could be mediated *via* anti-DT antibodies that are transferred to infants, because each of the 13 polysaccharides included in PCV13 is conjugated to CRM197 (a non-toxic mutant of DT). This is supported by the findings that maternal pre-existing anti-DT antibody levels were associated with lower immune response to serotypes 4, 6B and 9V after immunization with PCV7, and lower response to 19F (the only serotype conjugated to DT) after immunization with PCV10 (22). Our meta-analysis also showed enhanced immune response to tetanus and Hib vaccines conjugated to TT in infants born to women immunized with Tdap in pregnancy. Studies outside the setting of maternal immunization showed that immunization with Hib vaccine conjugated to TT was associated with higher anti-PRP levels when given concomitantly with meningococcal serogroup C polysaccharide vaccine conjugated to TT, supporting the enhancement of immunogenicity of Hib vaccines conjugated to TT (44).

This meta-analysis has a number of strengths. This is the first detailed and longitudinal analysis of the largest number of samples combined to establish the effect of immunization against pertussis in pregnancy on immune response to different vaccine antigens, routinely given to infants worldwide. Given the individual-participant nature of this meta-analysis, we were able to adjust for co-variates that could have affected the immune responses and to determine seroprotection rates for some vaccine-preventable diseases. Our study has some limitations. Most studies were conducted in high-income countries, thus data are less relevant to countries where whole-cell pertussis is being used for infants’ immunizations. As we investigated immune responses, our results should be interpreted in the context of clinical data.

Our meta-analysis quantified the effect of Tdap immunization in pregnancy on antigen-specific antibody responses to immunizations in infancy and showed inhibition of antibody responses to pertussis, diphtheria and some SPN serotypes. Continuous and enhanced surveillance is needed for multiple diseases (pertussis, diphtheria and IPD). Exploring the mechanism(s) of this modification of immune responses is critical and will help in informing design and use of futures vaccines to be used in pregnancy (e.g. conjugated group B *Streptococcus* and respiratory syncytial virus vaccines).

## Data Availability Statement

The datasets for this manuscript are not publicly available because of constraints based on the original consents provided by study participants. Requests to access the datasets should be directed to BA-R, (baburaya@bcchr.ubc.ca).

## Author Contributions

BA-R, KM, EL, and MS conceived and designed the study. BA-R and KM searched the literature, screened the articles for eligibility, and assessed the eligible articles for inclusion in the systematic review and meta-analysis, and the risk of bias of included studies. BA-R analyzed the data, produced the figures and wrote the first manuscript draft. BA-R, KM, FM, PZ, NC, SH, NR, DB, BH, BK, EL, and MS interpreted the data, critically reviewed, and edited the manuscript. All authors contributed to the article and approved the submitted version.

## Funding

The study was funded by British Columbia Immunization Committee.

## Conflict of Interest

BA-R is supported by the Canadian Health and Research Institute Vanier Canada Graduate scholarship. KM is the beneficiary of a postdoctoral mandate fellowship from the Fund for Scientific Research-Flanders (FWO 12R5819). MS is supported via salary awards from the BC Children’s Hospital Foundation, the Canadian Child Health Clinician Scientist Program and the Michael Smith Foundation for Health Research. MS has been an investigator on projects funded by GlaxoSmithKline, Merck, Pfizer, Sanofi Pasteur, Seqirus, Symvivo and VBI Vaccines. All funds have been paid to his institute, and he has not received any personal payments. SH has been an investigator on projects funded by GlaxoSmithKline, Merck, Pfizer, Sanofi-Pasteur, and CanSino; all funds have been paid to his University. SH has also served on ad hoc advisory boards for GSK, Sanofi, Pfizer, AsraZeneca, Merck, and IMV.

The remaining authors declare that the research was conducted in the absence of any commercial or financial relationships that could be construed as a potential conflict of interest.

## References

[1] Kilgore PE Salim AM Zervos MJ Schmitt HJ. Pertussis: Microbiology, Disease, Treatment, and Prevention. Clin Microbiol Rev (2016) 29:449–86. 10.1128/CMR.00083-15 PMC4861987 27029594

[2] Winter K Zipprich J Harriman K Murray EL Gornbein J Hammer SJ. Risk Factors Associated With Infant Deaths From Pertussis: A Case-Control Study. Clin Infect Dis (2015) 61:1099–106. 10.1093/cid/civ472 26082502

[3] Abu-Raya B Bettinger JA Vanderkooi OG Vaudry W Halperin SA Sadarangani M. Members of the Canadian Immunization Monitoring Program. 2020. Burden of Children Hospitalized With Pertussis in Canada in the Acellular Pertussis Vaccine Era, 1999-2015. J Pediatr Infect Dis Soc (2020) 9:118–27. 10.1093/jpids/piy128 PMC7192396 30535079

[4] Castagnini LA Munoz FM. Clinical Characteristics and Outcomes of Neonatal Pertussis: A Comparative Study. J Pediatr (2010) 156:498–500. 10.1016/j.jpeds.2009.10.013 20056236

[5] Amirthalingam G Andrews N Campbell H Ribeiro S Kara E Donegan K. Effectiveness of Maternal Pertussis Vaccination in England: An Observational Study. Lancet (2014) 384:1521–8. 10.1016/S0140-6736(14)60686-3 25037990

[6] Boulet SL Chamberlain AT Biswas HH Jamieson DJ. Trends in Infant Pertussis Hospitalizations in the United States, 2009-2017. JAMA (2019) 322:2134–6. 10.1001/jama.2019.15577 PMC6902169 31794618

[7] Romanin V Acosta AM Juarez MDV Briere E Sanchez SM Cordoba BL. Maternal Vaccination in Argentina: Tdap Vaccine Effectiveness During Pregnancy in Preventing Pertussis in Infants Less Than 2 Months of Age. Clin Infect Dis (2019) 70(3):380–7. 10.1093/cid/ciz217 PMC8876368 30877308

[8] Munoz FM Bond NH Maccato M Pinell P Hammill HA Swamy GK. Safety and Immunogenicity of Tetanus Diphtheria and Acellular Pertussis (Tdap) Immunization During Pregnancy in Mothers and Infants: A Randomized Clinical Trial. JAMA (2014) 311:1760–9. 10.1001/jama.2014.3633 PMC4333147 24794369

[9] Abu Raya B Srugo I Kessel A Peterman M Bader D Gonen R. The Effect of Timing of Maternal Tetanus, Diphtheria, and Acellular Pertussis (Tdap) Immunization During Pregnancy on Newborn Pertussis Antibody Levels - a Prospective Study. Vaccine (2014) 32:5787–93. 10.1016/j.vaccine.2014.08.038 25173476

[10] Van Savage J Decker MD Edwards KM Sell SH Karzon DT. Natural History of Pertussis Antibody in the Infant and Effect on Vaccine Response. J Infect Dis (1990) 161:487–92. 10.1093/infdis/161.3.487 2313127

[11] Englund JA Anderson EL Reed GF Decker MD Edwards KM Pichichero ME. The Effect of Maternal Antibody on the Serologic Response and the Incidence of Adverse Reactions After Primary Immunization With Acellular and Whole-Cell Pertussis Vaccines Combined With Diphtheria and Tetanus Toxoids. Pediatrics (1995) 96:580–4. 7659480

[12] Booy R Aitken SJ Taylor S Tudor-Williams G Macfarlane JA Moxon ER. Immunogenicity of Combined Diphtheria, Tetanus, and Pertussis Vaccine Given at 2, 3, and 4 Months Versus 3, 5, and 9 Months of Age. Lancet (1992) 339:507–10. 10.1016/0140-6736(92)90336-2 1346876

[13] Jones C Pollock L Barnett SM Battersby A Kampmann B. The Relationship Between Concentration of Specific Antibody At Birth and Subsequent Response to Primary Immunization. Vaccine (2014) 32:996–1002. 10.1016/j.vaccine.2013.11.104 24342250

[14] Maertens K Caboré RN Huygen K Hens N Van Damme P Leuridan E. Pertussis Vaccination During Pregnancy in Belgium: Results of a Prospective Controlled Cohort Study. Vaccine (2016) 34:142–50. 10.1016/j.vaccine.2015.10.100 26592142

[15] Maertens K Cabore RN Huygen K Vermeiren S Hens N Van Damme P. Pertussis Vaccination During Pregnancy in Belgium: Follow-Up of Infants Until 1 Month After the Fourth Infant Pertussis Vaccination at 15 Months of Age. Vaccine (2016) 34:3613–9. 10.1016/j.vaccine.2016.04.066 27142328

[16] Halperin SA Langley JM Ye L MacKinnon-Cameron D Elsherif M Allen VM. A Randomized Controlled Trial of the Safety and Immunogenicity of Tetanus, Diphtheria, and Acellular Pertussis Vaccine Immunization During Pregnancy and Subsequent Infant Immune Response. Clin Infect Dis (2018) 67:1063–71. 10.1093/cid/ciy244 30010773

[17] Stewart LA Clarke M Rovers M Riley RD Simmonds M Stewart G. Preferred Reporting Items for Systematic Review and Meta-Analyses of Individual Participant Data: The PRISMA-IPD Statement. JAMA (2015) 313:1657–65. 10.1001/jama.2015.3656 25919529

[18] Zimmermann P Curtis N. Factors That Influence the Immune Response to Vaccination. Clin Microbiol Rev (2019) 32:e00084–18. 10.1128/CMR.00084-18 PMC6431125 30867162

[19] Plotkin SA. Correlates of Protection Induced by Vaccination. Clin Vaccine Immunol (2010) 17:1055–65. 10.1128/CVI.00131-10 PMC2897268 20463105

[20] Sterne JA Hernán MA Reeves BC Savović J Berkman ND Viswanathan M. ROBINS-I: A Tool for Assessing Risk of Bias in Non-Randomised Studies of Interventions. BMJ (2016) 355:i4919. 10.1136/bmj.i4919 27733354 PMC5062054

[21] Barug D Pronk I van Houten MA Versteegh FGA Knol MJ van de Kassteele J. Maternal Pertussis Vaccination and Its Effects on the Immune Response of Infants Aged Up to 12 Months in the Netherlands: An Open-Label, Parallel, Randomised Controlled Trial. Lancet Infect Dis (2019) 19:392–401. 10.1016/S1473-3099(18)30717-5 30938299

[22] Barug D Berbers GAM van Houten MA Kuijer M Pronk I Knol MJ. Infant Antibody Levels Following 10-Valent Pneumococcal-Protein D Conjugate and Dtap-Hib Vaccinations in the First Year of Life After Maternal Tdap Vaccination: An Open-Label, Parallel, Randomised Controlled Trial. Vaccine (2020) 38:4632–9. 10.1016/j.vaccine.2020.04.001 32448624

[23] Hardy-Fairbanks AJ Pan SJ Decker MD Johnson DR Greenberg DP Kirkland KB. Immune Responses in Infants Whose Mothers Received Tdap Vaccine During Pregnancy. Pediatr Infect Dis J (2013) 32:1257–60. 10.1097/INF.0b013e3182a09b6a 23799518

[24] Hoang HTT Leuridan E Maertens K Nguyen TD Hens N Vu NH. Pertussis Vaccination During Pregnancy in Vietnam: Results of a Randomized Controlled Trial Pertussis Vaccination During Pregnancy. Vaccine (2016) 34:151–9. 10.1016/j.vaccine.2015.10.098 26529073

[25] Klein NP Abu-Elyazeed R Cheuvart B Janssens W Mesaros N. Immunogenicity and Safety Following Primary and Booster Vaccination With a Hexavalent Diphtheria, Tetanus, Acellular Pertussis, Hepatitis B, Inactivated Poliovirus and Haemophilus Influenzae Type B Vaccine: A Randomized Trial in the United States. Hum Vaccin Immunother (2019) 15:809–21. 10.1080/21645515.2018.1549449 PMC6605854 30444673

[26] Ladhani SN Andrews NJ Southern J Jones CE Amirthalingam G Waight PA. Antibody Responses After Primary Immunization in Infants Born to Women Receiving a Pertussis-Containing Vaccine During Pregnancy: Single Arm Observational Study With a Historical Comparator. Clin Infect Dis (2015) 61:1637–44. 10.1093/cid/civ695 26374816

[27] Maertens K Hoang TT Nguyen TD Cabore RN Duong TH Huygen K. The Effect of Maternal Pertussis Immunization on Infant Vaccine Responses to a Booster Pertussis-Containing Vaccine in Vietnam. Clin Infect Dis (2016) 63:S197–204. 10.1093/cid/ciw551 27838673 PMC5106623

[28] Maertens K Burbidge P Van Damme P Goldblatt D Leuridan E. Pneumococcal Immune Response in Infants Whose Mothers Received Tetanus, Diphtheria and Acellular Pertussis Vaccination During Pregnancy. Pediatr Infect Dis J (2017) 36:1186–92. 10.1097/INF.0000000000001601 28399054

[29] Orije MR Corbière V Maertens K Mahieu L Van Damme P Cools N. Vancouver: Presented at the International Neonatal & Maternal Immunization Symposium (2019).

[30] Perrett KP Halperin SA Nolan T Carmona Martínez A Martinón-Torres F García-Sicilia J. Impact of Tetanus-Diphtheria-Acellular Pertussis Immunization During Pregnancy on Subsequent Infant Immunization Seroresponses: Follow-Up From a Large Randomized Placebo-Controlled Trial. Vaccine (2019) 38(8):2105–114. 10.1016/j.vaccine.2019.10.104 31776027

[31] Rice TF Diavatopoulos DA Smits GP van Gageldonk PGM Berbers GAM van der Klis FR. Antibody Responses to Bordetella Pertussis and Other Childhood Vaccines in Infants Born to Mothers Who Received Pertussis Vaccine in Pregnancy - A Prospective, Observational Cohort Study From the United Kingdom. Clin Exp Immunol (2019) 197:1–10. 10.1111/cei.13275 30758857 PMC6591149

[32] Zimmermann P Perrett KP Messina NL Donath S Ritz N van der Klis FRM. The Effect of Maternal Immunisation During Pregnancy on Infant Vaccine Responses. EClinicalMedicine (2019) 13:21–30. 10.1016/j.eclinm.2019.06.010 31517260 PMC6733996

[33] Amirthalingam G Campbell H Ribeiro S Fry NK Ramsay M Miller E. Sustained Effectiveness of the Maternal Pertussis Immunization Program in England 3 Years Following Introduction. Clin Infect Dis (2016) 63:S236–43. 10.1093/cid/ciw559 PMC5106626 27838678

[34] Baxter R Bartlett J Fireman B Lewis E Klein NP. Effectiveness of Vaccination During Pregnancy to Prevent Infant Pertussis. Pediatrics (2017) 139. 10.1542/peds.2016-4091 28557752

[35] Abu-Raya B Edwards KM. Interference With Pertussis Vaccination in Infants After Maternal Pertussis Vaccination. Pediatrics (2020) 146. 10.1542/peds.2019-3579 32753370

[36] Kim D Huey D Oglesbee M Niewiesk S. Insights Into the Regulatory Mechanism Controlling the Inhibition of Vaccine-Induced Seroconversion by Maternal Antibodies. Blood (2011) 117:6143–51. 10.1182/blood-2010-11-320317 PMC3122939 21357766

[37] Vono M Eberhardt CS Auderset F Mastelic-Gavillet B Lemeille S Christensen D. Maternal Antibodies Inhibit Neonatal and Infant Responses to Vaccination by Shaping the Early-Life B Cell Repertoire Within Germinal Centers. Cell Rep (2019) 28:1773–84.e1775. 10.1016/j.celrep.2019.07.047 31412246

[38] Niewiesk S. Maternal Antibodies: Clinical Significance, Mechanism of Interference With Immune Responses, and Possible Vaccination Strategies. Front Immunol (2014) 5:446. 10.3389/fimmu.2014.00446 25278941 PMC4165321

[39] Clarke KEN MacNeil A Hadler S Scott C Tiwari TSP Cherian T. Global Epidemiology of Diphtheria, 2000-2017. Emerg Infect Dis (2019) 25:1834–42. 10.3201/eid2510.190271 PMC6759252 31538559

[40] Centers for Disease Control and Prevention. Diphtheria. (2020). Available at: https://www.cdc.gov/diphtheria/about/index.html (Accessed December 10th).

[41] Andrews NJ Waight PA Burbidge P Pearce E Roalfe L Zancolli M. Serotype-Specific Effectiveness and Correlates of Protection for the 13-Valent Pneumococcal Conjugate Vaccine: A Postlicensure Indirect Cohort Study. Lancet Infect Dis (2014) 14:839–46. 10.1016/S1473-3099(14)70822-9 25042756

[42] Voysey M Fanshawe TR Kelly DF O’Brien KL Kandasamy R Shrestha S. Serotype-Specific Correlates of Protection for Pneumococcal Carriage: An Analysis of Immunity in 19 Countries. Clin Infect Dis (2018) 66:913–20. 10.1093/cid/cix895 29069415

[43] Goldblatt D Southern J Andrews NJ Burbidge P Partington J Roalfe L. Pneumococcal Conjugate Vaccine 13 Delivered as One Primary and One Booster Dose (1â ˆ+Â ˆ1) Compared With Two Primary Doses and a Booster (2â ˆ+Â ˆ1) in UK Infants: A Multicentre, Parallel Group Randomised Controlled Trial. Lancet Infect Dis (2018) 18:171–9. 10.1016/S1473-3099(17)30654-0 PMC5805912 29174323

[44] Kitchin NRE Southern J Morris R Hemme F Thomas S Watson MW. Evaluation of a Diphtheria-Tetanus-Acellular Pertussis-Inactivated Poliovirus-Haemophilus Influenzae Type B Vaccine Given Concurrently With Meningococcal Group C Conjugate Vaccine at 2, 3 and 4 Months of Age. Arch Dis Childhood (2007) 92:11–6. 10.1136/adc.2005.076109 PMC2083161 16670121

