# Supplementary material for: The Effect of Tetanus-Diphtheria-Acellular-Pertussis Immunization During Pregnancy on Infant Antibody Responses: Individual-Participant Data Meta-Analysis
Source: Front Immunol. 2021 Jul 6;12:689394. doi: 10.3389/fimmu.2021.689394 (PMC8299947; doi:10.3389/fimmu.2021.689394)
Supplement: Supplementary file 1 [file DataSheet_1.docx]

**Supplementary appendix**

**Supplementary Table 1:** PRISMA-IPD Checklist of items to include when reporting a systematic review and meta-analysis of individual participant data (IPD)

**Supplementary Methods:** Systematic literature search procedures

**Supplementary Table 2:** Risk of bias assessment of randomized controlled trials.

**Supplementary Table 3:** Risk of bias assessment of non-randomized studies.

**Supplementary Table 1: PRISMA-IPD Checklist of items to include when reporting a systematic review and meta-analysis of individual participant data (IPD)**

| **PRISMA-IPD**  **Section/topic** | **Item No** | **Checklist item** | **Reported on page** |
| --- | --- | --- | --- |
| **Title** | | | |
| Title | 1 | Identify the report as a systematic review and meta-analysis of individual participant data. | 1 |
| **Abstract** | | | |
| Structured summary | 2 | Provide a structured summary including as applicable: | 3-4 |
|  |  | **Background**: state research question and main objectives, with information on participants, interventions, comparators and outcomes. |  |
|  |  | **Methods**: report eligibility criteria; data sources including dates of last bibliographic search or elicitation, noting that IPD were sought; methods of assessing risk of bias. |  |
|  |  | **Results**: provide number and type of studies and participants identified and number (%) obtained; summary effect estimates for main outcomes (benefits and harms) with confidence intervals and measures of statistical heterogeneity. Describe the direction and size of summary effects in terms meaningful to those who would put findings into practice. |  |
|  |  | **Discussion:** state main strengths and limitations of the evidence, general interpretation of the results and any important implications. |  |
|  |  | **Other:** report primary funding source, registration number and registry name for the systematic review and IPD meta-analysis. |  |
| **Introduction** | | | |
| Rationale | 3 | Describe the rationale for the review in the context of what is already known. | 5-6 |
| Objectives | 4 | Provide an explicit statement of the questions being addressed with reference, as applicable, to participants, interventions, comparisons, outcomes and study design (PICOS). Include any hypotheses that relate to particular types of participant-level subgroups. | 6 |
| **Methods** | | | |
| Protocol and registration | 5 | Indicate if a protocol exists and where it can be accessed. If available, provide registration information including registration number and registry name. Provide publication details, if applicable. | Page 10 and Supplementary page 6 |
| Eligibility criteria | 6 | Specify inclusion and exclusion criteria including those relating to participants, interventions, comparisons, outcomes, study design and characteristics (e.g. years when conducted, required minimum follow-up). Note whether these were applied at the study or individual level i.e. whether eligible participants were included (and ineligible participants excluded) from a study that included a wider population than specified by the review inclusion criteria. The rationale for criteria should be stated. | 7-8 |
| Identifying studies - information sources | 7 | Describe all methods of identifying published and unpublished studies including, as applicable: which bibliographic databases were searched with dates of coverage; details of any hand searching including of conference proceedings; use of study registers and agency or company databases; contact with the original research team and experts in the field; open adverts and surveys. Give the date of last search or elicitation. | Supplementary page 6 |
| Identifying studies - search | 8 | Present the full electronic search strategy for at least one database, including any limits used, such that it could be repeated. | Supplementary page 6 |
| Study selection processes | 9 | State the process for determining which studies were eligible for inclusion. | Supplementary page 6 |
| Data collection processes | 10 | Describe how IPD were requested, collected and managed, including any processes for querying and confirming data with investigators. If IPD were not sought from any eligible study, the reason for this should be stated (for each such study). | Figure 1 |
|  |  | If applicable, describe how any studies for which IPD were not available were dealt with. This should include whether, how and what aggregate data were sought or extracted from study reports and publications (such as extracting data independently in duplicate) and any processes for obtaining and confirming these data with investigators. |  |
| Data items | 11 | Describe how the information and variables to be collected were chosen. List and define all study level and participant level data that were sought, including baseline and follow-up information. If applicable, describe methods of standardising or translating variables within the IPD datasets to ensure common scales or measurements across studies. | Pages 9-10 |
| IPD integrity | A1 | Describe what aspects of IPD were subject to data checking (such as sequence generation, data consistency and completeness, baseline imbalance) and how this was done. | Page 12 |
| Risk of bias assessment in individual studies. | 12 | Describe methods used to assess risk of bias in the individual studies and whether this was applied separately for each outcome. If applicable, describe how findings of IPD checking were used to inform the assessment. Report if and how risk of bias assessment was used in any data synthesis. | Page 10 |
| Specification of outcomes and effect measures | 13 | State all treatment comparisons of interests. State all outcomes addressed and define them in detail. State whether they were pre-specified for the review and, if applicable, whether they were primary/main or secondary/additional outcomes. Give the principal measures of effect (such as risk ratio, hazard ratio, difference in means) used for each outcome. | Pages 9-10 |
| Synthesis methods | 14 | Describe the meta-analysis methods used to synthesise IPD. Specify any statistical methods and models used. Issues should include (but are not restricted to):   - Use of a one-stage or two-stage approach. - How effect estimates were generated separately within each study and combined across studies (where applicable). - Specification of one-stage models (where applicable) including how clustering of patients within studies was accounted for. - Use of fixed or random effects models and any other model assumptions, such as proportional hazards. - How (summary) survival curves were generated (where applicable). - Methods for quantifying statistical heterogeneity (such as I^2^ and τ^2^). - How studies providing IPD and not providing IPD were analysed together (where applicable). - How missing data within the IPD were dealt with (where applicable). | Pages 9-10 |
| Exploration of variation in effects | A2 | If applicable, describe any methods used to explore variation in effects by study or participant level characteristics (such as estimation of interactions between effect and covariates). State all participant-level characteristics that were analysed as potential effect modifiers, and whether these were pre-specified. | Not applicable |
| Risk of bias across studies | 15 | Specify any assessment of risk of bias relating to the accumulated body of evidence, including any pertaining to not obtaining IPD for particular studies, outcomes or other variables. | Not applicable |
| Additional analyses | 16 | Describe methods of any additional analyses, including sensitivity analyses. State which of these were pre-specified. | Not applicable |
| **Results** | | | |
| Study selection and IPD obtained | 17 | Give numbers of studies screened, assessed for eligibility, and included in the systematic review with reasons for exclusions at each stage. Indicate the number of studies and participants for which IPD were sought and for which IPD were obtained. For those studies where IPD were not available, give the numbers of studies and participants for which aggregate data were available. Report reasons for non-availability of IPD. Include a flow diagram. | Page 12, Figure 1 |
| Study characteristics | 18 | For each study, present information on key study and participant characteristics (such as description of interventions, numbers of participants, demographic data, unavailability of outcomes, funding source, and if applicable duration of follow-up). Provide (main) citations for each study. Where applicable, also report similar study characteristics for any studies not providing IPD. | Table 1 |
| IPD integrity | A3 | Report any important issues identified in checking IPD or state that there were none. | Page 12 |
| Risk of bias within studies | 19 | Present data on risk of bias assessments. If applicable, describe whether data checking led to the up-weighting or down-weighting of these assessments. Consider how any potential bias impacts on the robustness of meta-analysis conclusions. | Page 12, Supplementary Table 1 and 2 |
| Results of individual studies | 20 | For each comparison and for each main outcome (benefit or harm), for each individual study report the number of eligible participants for which data were obtained and show simple summary data for each intervention group (including, where applicable, the number of events), effect estimates and confidence intervals. These may be tabulated or included on a forest plot. | Not applicable |
| Results of syntheses | 21 | Present summary effects for each meta-analysis undertaken, including confidence intervals and measures of statistical heterogeneity. State whether the analysis was pre-specified, and report the numbers of studies and participants and, where applicable, the number of events on which it is based. | Pages 13-18 |
|  |  | When exploring variation in effects due to patient or study characteristics, present summary interaction estimates for each characteristic examined, including confidence intervals and measures of statistical heterogeneity. State whether the analysis was pre-specified. State whether any interaction is consistent across trials. |  |
|  |  | Provide a description of the direction and size of effect in terms meaningful to those who would put findings into practice. |  |
| Risk of bias across studies | 22 | Present results of any assessment of risk of bias relating to the accumulated body of evidence, including any pertaining to the availability and representativeness of available studies, outcomes or other variables. | Supplementary Table 1 and 2 |
| Additional analyses | 23 | Give results of any additional analyses (e.g. sensitivity analyses). If applicable, this should also include any analyses that incorporate aggregate data for studies that do not have IPD. If applicable, summarise the main meta-analysis results following the inclusion or exclusion of studies for which IPD were not available. | Table 2 |
| **Discussion** | | | |
| Summary of evidence | 24 | Summarise the main findings, including the strength of evidence for each main outcome. | Page 19-23 |
| Strengths and limitations | 25 | Discuss any important strengths and limitations of the evidence including the benefits of access to IPD and any limitations arising from IPD that were not available. | Page 22 |
| Conclusions | 26 | Provide a general interpretation of the findings in the context of other evidence. | Page 23 |
| Implications | A4 | Consider relevance to key groups (such as policy makers, service providers and service users). Consider implications for future research. | Page 23 |
| **Funding** | | | |
| Funding | 27 | Describe sources of funding and other support (such as supply of IPD), and the role in the systematic review of those providing such support. | Page 25 |

**A1 – A3 denote new items that are additional to standard PRISMA items. A4 has been created as a result of re-arranging content of the standard PRISMA statement to suit the way that systematic review IPD meta-analyses are reported.**

© Reproduced with permission of the PRISMA IPD Group, which encourages sharing and reuse for non-commercial purposes

**Supplementary Methods: Systematic literature search procedures**

**Data sources and search strategy**

PubMed, MEDLINE, Embase, CINAHL, and the Cochrane Central Register of Controlled Trials (CENTRAL) databases were searched for English literature reporting immunoglobulin G (IgG) levels following primary and booster immunizations in infants born to women immunized against pertussis in pregnancy and infants of women unimmunized in pregnancy, published between January 1^st^, 1990 and January 6^th^, 2020. Original search performed on February 21^st^, 2017 and updated searches performed on June 4^th^, 2018 and January 6^th^, 2020). The following search terms were used: "pertussis immunization" OR "pertussis vaccination" OR "Tdap vaccination" OR "Tdap vaccine" OR "Tdap immunization" OR “Tdap pregnancy” AND “interference” OR “antibody response” OR “immunogenicity” OR “immune responses” AND “pregnancy". Additional studies were identified by searching reference lists of identified publications, trial registries (clinicaltrials.gov) and reviewing abstracts presented at conferences (International Neonatal & Maternal Immunization Symposium and European Society for Paediatric Infectious Diseases 2019). Randomized and non-randomized studies were included. Letters, editorials and review articles containing no primary data were excluded.

The systematic literature search was performed by two independent researchers (BA and KM) and references were de-duplicated automatically by EndNote^TM^ and manually according to the last name of the first author. Remaining references were screened by title and abstract by two researchers (BA and KM), with a third researcher (MS) consulted when necessary. Articles screened and found to be potentially eligible were fully assessed (full-text assessed against inclusion and exclusion criteria) by two researchers (BA and KM) with a third researcher (MS) consulted when necessary.

**Supplementary Table 2: Risk of bias assessment of randomized controlled trials.**

| **Domain** | **Selection bias** | | **Performance bias.** | **Detection bias.** | **Attrition bias.** | **Reporting bias.** | **Other bias.** |
| --- | --- | --- | --- | --- | --- | --- | --- |
|  | **Random sequence generation** | **Allocation concealment.** | **Blinding of participants and personnel *** | **Blinding of outcome assessment**** | **Incomplete outcome data** | **Selective reporting.** | **Other sources of bias** |
| Munoz et al^1^ | Low | Low | Low | Low | Low | Low |  |
| Hoang et al ^2^ | Low | Low | Low | Low | Low | Low |  |
| Maertens et al^3^ | Low | Low | Low | Low | Low | Low |  |
| Halperin et al^4^ | Low | Low | Low | Low | Low | Low | High *** |
| Barug et al^5^ | Low | Low | Low | Low | Low | Low |  |
| Perret et al ^6^ | Low | Low | Low | Low | Low | Low | High*** |
| Barug et al^7^ | Low | Low | Low | Low | Low | Low |  |

Risk of bias assessed against the Cochrane Risk of Bias tool for RCTs Cochrane Handbook for Systematic Reviews of Interventions Version 5.1.0, chapter 8, tables 8.5.a)). For each domain assessed, options included: ‘low risk’, ‘high risk, or ‘unclear risk’, with the last category indicating either lack of information or uncertainty over the potential for bias.

*As the outcomes evaluated in this meta-analysis are antibody levels, blinding of participants and personnel (other than outcome assessors [laboratory personnel]) was not deemed to bias the performance. Thus, a “low risk” was deemed appropriate for all the studies.

**This was assessed against blinding of laboratory personnel to the intervention arm.

*** Industry funded studies.

**Supplementary Table 3: Risk of bias assessment of non-randomized studies.**

| **Domain** | **Pre-intervention domains** | | **At-intervention domain** | **Post-intervention domains** | | | |
| --- | --- | --- | --- | --- | --- | --- | --- |
|  | **Bias due to confounding** | **Bias in selection of participants into the study** | **Bias in classification of interventions** | **Bias due to deviations from intended interventions** | **Bias due to missing data** | **Bias in measurement of outcomes** | **Bias in selection of the reported result** |
| Hardy-Fairbanks et al^8^* | Critical | Low | Low | Low | Moderate | Low | Low |
| Ladhani et al^9^* | Low | Low | Low | Low | Low | Low | Low |
| Maertens et al^10^ | Low | Low | Low | Low | Low | Low | Low |
| Maertens et al^11^ | Low | Low | Low | Low | Low | Low | Low |
| Maertens et al^12^ | Low | Low | Low | Low | Low | Low | Low |
| Rice et al^13^ | Low | Low | Low | Low | Low | Low | Low |
| Klein et al ^14^** | NI | NI | Low | Low | Low | Low | Low |
| Zimmermann^15^*** | Low | Low | Low | Low | Low | Low | Low |
| Maertens et al^16^ | Low | Low | Low | Low | Low | Low | Low |

Assessment was based on Sterne JA et al. [ROBINS-I: a tool for assessing risk of bias in non-randomised studies of interventions.](https://pubmed.ncbi.nlm.nih.gov/27733354/) BMJ 2016;355:i4919 . For each domain assessed, options included: Low/Moderate/Serious/Critical/No information (NI).

* Not included in the individual-participant-data meta-analysis

** The original study randomized infants to receive different immunization schedules. For the purpose of this meta-analysis, women were not randomized to receive tetanus-diphtheria-acellular-pertussis vaccine or not in pregnancy.

*** The original study randomized infants to receive or not BCG at birth. For the purpose of this meta-analysis, women were not randomized to receive tetanus-diphtheria-acellular-pertussis vaccine or not in pregnancy

**References:**

1. Munoz FM, Bond NH, Maccato M, et al. Safety and immunogenicity of tetanus diphtheria and acellular pertussis (Tdap) immunization during pregnancy in mothers and infants: a randomized clinical trial. *JAMA* 2014; **311**(17): 1760-9.

2. Hoang HT, Leuridan E, Maertens K, et al. Pertussis vaccination during pregnancy in Vietnam: Results of a randomized controlled trial Pertussis vaccination during pregnancy. *Vaccine* 2016; **34**(1): 151-9.

3. Maertens K, Hoang TT, Nguyen TD, et al. The Effect of Maternal Pertussis Immunization on Infant Vaccine Responses to a Booster Pertussis-Containing Vaccine in Vietnam. *Clin Infect Dis* 2016; **63**(suppl 4): S197-s204.

4. Halperin SA, Langley JM, Ye L, et al. A Randomized Controlled Trial of the Safety and Immunogenicity of Tetanus, Diphtheria, and Acellular Pertussis Vaccine Immunization During Pregnancy and Subsequent Infant Immune Response. *Clin Infect Dis* 2018; **67**(7): 1063-71.

5. Barug D, Pronk I, van Houten MA, et al. Maternal pertussis vaccination and its effects on the immune response of infants aged up to 12 months in the Netherlands: an open-label, parallel, randomised controlled trial. *Lancet Infect Dis* 2019; **19**(4): 392-401.

6. Perrett KP, Halperin SA, Nolan T, et al. Impact of tetanus-diphtheria-acellular pertussis immunization during pregnancy on subsequent infant immunization seroresponses: follow-up from a large randomized placebo-controlled trial. *Vaccine* 2019.

7. Barug D, Berbers GAM, van Houten MA, et al. Infant antibody levels following 10-valent pneumococcal-protein D conjugate and DTaP-Hib vaccinations in the first year of life after maternal Tdap vaccination: An open-label, parallel, randomised controlled trial. *Vaccine* 2020; **38**(29): 4632-9.

8. Hardy-Fairbanks AJ, Pan SJ, Decker MD, et al. Immune responses in infants whose mothers received Tdap vaccine during pregnancy. *Pediatr Infect Dis J* 2013; **32**(11): 1257-60.

9. Ladhani SN, Andrews NJ, Southern J, et al. Antibody responses after primary immunization in infants born to women receiving a pertussis-containing vaccine during pregnancy: single arm observational study with a historical comparator. *Clin Infect Dis* 2015; **61**(11): 1637-44.

10. Maertens K, Caboré RN, Huygen K, Hens N, Van Damme P, Leuridan E. Pertussis vaccination during pregnancy in Belgium: Results of a prospective controlled cohort study. *Vaccine* 2016; **34**(1): 142-50.

11. Maertens K, Cabore RN, Huygen K, et al. Pertussis vaccination during pregnancy in Belgium: Follow-up of infants until 1 month after the fourth infant pertussis vaccination at 15 months of age. *Vaccine*; **34**(31): 3613-9.

12. Maertens K, Burbidge P, Van Damme P, Goldblatt D, Leuridan E. Pneumococcal Immune Response in Infants Whose Mothers Received Tetanus, Diphtheria and Acellular Pertussis Vaccination During Pregnancy. *Pediatr Infect Dis J* 2017; **36**(12): 1186-92.

13. Rice TF, Diavatopoulos DA, Smits GP, et al. Antibody responses to Bordetella pertussis and other childhood vaccines in infants born to mothers who received pertussis vaccine in pregnancy - a prospective, observational cohort study from the United Kingdom. *Clin Exp Immunol* 2019; **197**(1): 1-10.

14. Klein NP, Abu-Elyazeed R, Cheuvart B, Janssens W, Mesaros N. Immunogenicity and safety following primary and booster vaccination with a hexavalent diphtheria, tetanus, acellular pertussis, hepatitis B, inactivated poliovirus and Haemophilus influenzae type b vaccine: a randomized trial in the United States. *Hum Vaccin Immunother* 2019; **15**(4): 809-21.

15. Zimmermann P, Perrett KP, Messina NL, et al. The Effect of Maternal Immunisation During Pregnancy on Infant Vaccine Responses. *EClinicalMedicine* 2019; **13**: 21-30.

16. Orije MR , Corbière V, Maertens K, Mahieu L, Van Damme P, Cools N, Mascart F, Leuridan E. The effect of pertussis vaccination during pregnancy on the immune responses after primary and booster vaccination in term and preterm born infants . Presented at the   International Neonatal & Maternal Immunization Symposium, Vancouver, 2019.  .
